# A qualitative systematic review and thematic synthesis exploring the impacts of clinical academic activity by healthcare professionals outside medicine

**DOI:** 10.1186/s12913-021-06354-y

**Published:** 2021-04-29

**Authors:** Lisa Newington, Mary Wells, Adine Adonis, Lee Bolton, Layla Bolton Saghdaoui, Margaret Coffey, Jennifer Crow, Olga Fadeeva Costa, Catherine Hughes, Matthew Savage, Lillie Shahabi, Caroline M. Alexander

**Affiliations:** 1 Imperial College Healthcare NHS Trust, Education Centre, Charing Cross Hospital, Fulham Palace Road, London, W6 8RF UK; 2 Department of Surgery and Cancer, Faculty of Medicine, Imperial College London, London, UK; 3 Department of Brain Sciences, Faculty of Medicine, Imperial College London, London, UK

**Keywords:** Research impact, Clinical academics, Allied health professions, Nursing, Midwifery, Systematic review, Thematic synthesis

## Abstract

**Background:**

There are increasing opportunities for healthcare professionals outside medicine to be involved in and lead clinical research. However, there are few roles within these professions that include time for research. In order to develop such roles, and evaluate effective use of this time, the range of impacts of this clinical academic activity need to be valued and understood by healthcare leaders and managers. To date, these impacts have not been comprehensively explored, but are suggested to extend beyond traditional quantitative impact metrics, such as publications, citations and funding awards.

**Methods:**

Ten databases, four grey literature repositories and a naïve web search engine were systematically searched for articles reporting impacts of clinical academic activity by healthcare professionals outside medicine. Specifically, this did not include the direct impacts of the research findings, rather the impacts of the research activity. All stages of the review were performed by a minimum of two reviewers and reported impacts were categorised qualitatively according to a modified VICTOR (making Visible the ImpaCT Of Research) framework.

**Results:**

Of the initial 2704 identified articles, 20 were eligible for inclusion. Identified impacts were mapped to seven themes: impacts for patients; impacts for the service provision and workforce; impacts to research profile, culture and capacity; economic impacts; impacts on staff recruitment and retention; impacts to knowledge exchange; and impacts to the clinical academic.

**Conclusions:**

Several overlapping sub-themes were identified across the main themes. These included the challenges and benefits of balancing clinical and academic roles, the creation and implementation of new evidence, and the development of collaborations and networks. These may be key areas for organisations to explore when looking to support and increase academic activity among healthcare professionals outside medicine. The modified VICTOR tool is a useful starting point for individuals and organisations to record the impact of their research activity. Further work is needed to explore standardised methods of capturing research impact that address the full range of impacts identified in this systematic review and are specific to the context of clinical academics outside medicine.

**Supplementary Information:**

The online version contains supplementary material available at 10.1186/s12913-021-06354-y.

## Background

There is compelling evidence that research active healthcare organisations have improved care performance compared to their non-research active counterparts [1]. Examples include patients feeling better informed about their condition and medication, having greater confidence in their healthcare staff [2], greater staff adherence to treatment guidelines [3] and lower mortality rates [3, 4]. In the UK, this has resulted in correlation between research activity and the national healthcare inspection rating [4].

Traditionally, healthcare research has been associated with medical professionals (doctors), with approximately 5% of UK medical consultants working in clinical academic roles [5, 6]. Clinical academics engage in clinical practice and also conduct and lead programmes of applied health and/or social care research, often directly aimed at improving patient care and care pathways [7].

Healthcare professionals outside medicine are increasingly developing the expertise to lead clinically relevant research, with the aim of 1% of this workforce being employed in clinical academic roles by 2030 [8]. Healthcare professions outside medicine include: nursing, midwifery, the allied health professions (art therapists, dietitians, drama therapists, music therapists, occupational therapists, orthoptists, operating department practitioners, osteopaths, podiatrists, prosthetists/orthotists, paramedics, physiotherapists, radiographers, and speech and language therapists), clinical psychologists, healthcare scientists and pharmacists. Within the UK, the drive to increase the clinical academic workforce is supported by a targeted Health Education England/National Institute for Health Research funding stream specifically for these professions [9], and through fellowship funding from a number of national health charities. Similar schemes exist elsewhere [10–13].

As clinical academic activity increases, there is a need to evaluate its impact at both individual and organisational levels, and across the short to longer term. Several frameworks have been designed to guide impact assessments for healthcare research and these have recently been systematically reviewed to create a summary framework [14]. However, the focus is on evaluating individual programmes of research, rather than the impact of collective research activity within an organisation. This is also the case with other research impact assessment tools [15].

Outside the medical professions, the impacts of dedicated allied health professional (AHP) research roles have been systematically reviewed to explore their outcomes in terms of building research capacity and culture. Wenke and Mickan described varied roles, but most often these centred on the development of researchers’ own research projects and their dissemination [10]. Additional responsibilities included supervising others and developing strategies to promote research activity. These roles were found to have positive impacts on individual research skills, research outputs and research culture, however other areas of impact were not assessed, such as patient outcomes, changes to clinical training or practice guidelines, or increased investment. Importantly, only one study described practising clinicians with dual clinical and research roles, and other non-medical professionals, such as nurses and midwives, were excluded from the review.

Existing reviews have only included published research studies, thus overlooking impact reports that have been compiled by individual healthcare organisations or collaborations [16, 17]. Such documents contain valuable insights and reflections on clinical academic programmes, often in the form of case studies, and are useful for other healthcare providers supporting or developing their own clinical academic strategies.

The current systematic review was developed in order to understand the full range of impacts of non-medical clinical academic roles. A cross-disciplinary approach was taken to include clinical academic activity among nurses, midwives, AHPs and other non-medical healthcare professionals.

## Methods

The review protocol was pre-registered with the Open Science Foundation [18] and followed the PRISMA protocol reporting guidelines [19]. The primary review question was: what are the reported impacts of clinical academic activity among practising healthcare professionals outside medicine?

### Selection criteria

Impact was not pre-defined for the purposes of this review, and eligible articles were those reporting any form of impact that was attributed to clinical academic activity carried out by non-medical healthcare professionals. This did not include the reported outcomes of clinical research studies, rather the impact of these individuals being involved in research activity. Clinical academic activity was defined as the review intervention, and was the involvement of practising clinicians in research. This included specific research roles, such as research fellowships or combined clinical academic positions, in addition to other protected research time or opportunities to be involved in research. The review population was defined as healthcare professionals outside medicine. Full eligibility criteria, including the list of eligible healthcare professions, are provided in Table 1.

**Table 1:** Review inclusion and exclusion criteria

INCLUSION CRITERIA	EXCLUSION CRITERIA
***Non-medical healthcare professionals*** This included: nurses; midwives; allied health professionals (art therapists, dietitians, drama therapists, music therapists, occupational therapists, orthoptists, operating department practitioners, osteopaths, podiatrists, prosthetists/orthotists, paramedics, physiotherapists, radiographers, and speech and language therapists); clinical psychologists; healthcare scientists and pharmacists. Assistants, technicians and support workers for these professions were also included.	***Doctors and dentists*** Mixed research teams involving medical and non-medical healthcare professionals were excluded unless data were reported separately for the non-medical healthcare professionals.
***Clinical academic activity*** Involvement of practising clinicians in clinical research. This included specific research roles, protected research time and other opportunities to be involved in research.	***Audit and service evaluation*** Both are routinely required components of clinical roles and were therefore not defined as clinical academic activity. ***Pure academic or educational research*** Research based in a higher education institute without impact on healthcare organisations or the staff working in these organisation.
***Impact of clinical academic activity*** The types of impact were not pre-defined and could include the assessment of clinical, economic, workforce or other outcomes that were attributed to the clinical academic activity.	***Report clinical research outcomes only*** Studies reporting the outcome of clinical research questions, rather than the impact of the research activity, were excluded.
***Studies reporting quantitative and/or qualitative primary data and systematic reviews*** Where eligible systematic reviews were identified, their primary papers were included and screened separately.	***Opinion pieces and non-systematic reviews of the literature***
***Published after 2000*** To identify the impact of clinical academic activity in the context of current and recent past practice, the review was restricted to the past 20 years.	***Published before 2000*** Due to rapidly evolving healthcare environments, it was felt that such articles would not represent current practice.

### Search strategy

**Fig. 1:**
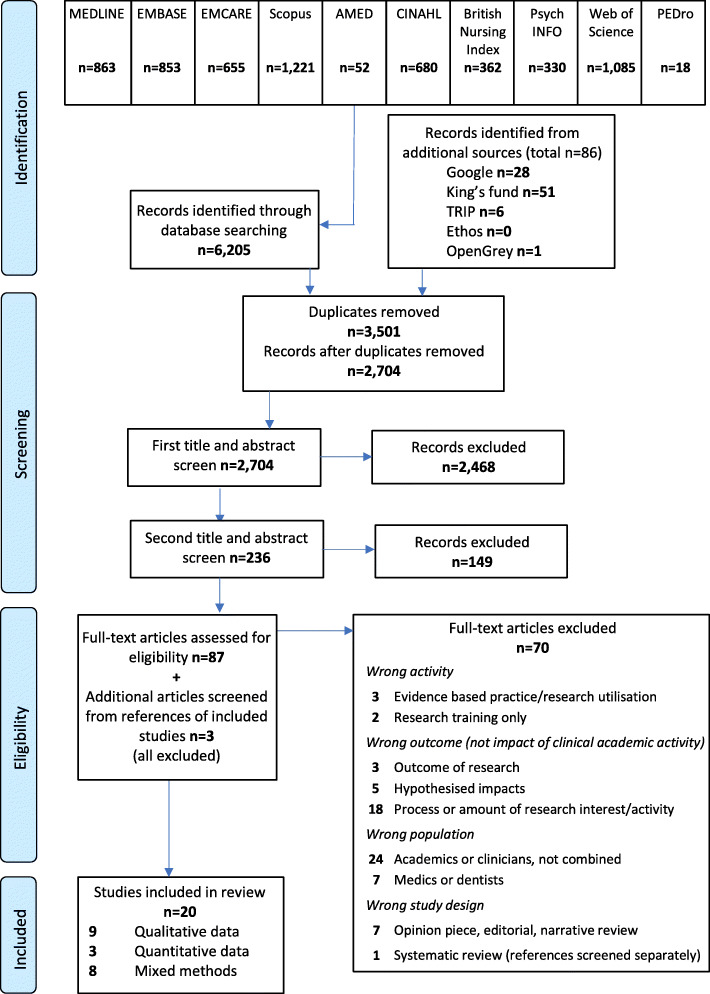
PRISMA flowchart

Ten healthcare databases and four grey literature repositories were individually searched by the lead author between December 2019 and January 2020. Search locations are shown in Fig. 1. The example search strategy for Medline is provided in Additional File 1, which included i) terms for the different non-medical clinical disciplines, and ii) terms for clinical academic activity, combined with iii) terms for impact. The search strategy was developed and piloted by LN, CMA and MW, with additional assistance from a healthcare librarian (LG). There were no restrictions for country of publication, but due to time and resource limitations, articles were restricted to those available in the English language. An additional Google search for ‘impact of clinical academic nursing, midwifery and allied health’ was conducted in a naïve browser and the first 50 hits recorded.

### Eligibility assessment

Identified references were exported into Covidence (Covidence.org) and duplicates removed. Title and abstract screening were performed in two stages. Firstly, the lead author and two members of the review team (JC and OFC) independently screened out those articles which clearly met the exclusion criteria. Any disagreements were resolved by discussion. Secondly, the lead author and two members of the review team (LS and LB) independently screened the remaining articles against both the inclusion and exclusion criteria. Again, any disagreements were resolved by discussion. Full text screening was independently performed by LN and one of five members of the review team (AA, MC, LS, OFC and LBS); disagreements were resolved by MW and CMA.

### Quality assessment

The mixed methods appraisal tool (MMAT) was used to evaluate the quality of the included studies [20]. The assessment form was piloted prior to use and modified to include key components of the qualitative checklist proposed by Walsh et al. [21]. The quality assessment form template is provided in Additional File 2. Quality assessment was conducted independently by LN and one of eight members of the review team (AA, LBS, CL, LS, MC, JJ, LB and JC). Disagreements were resolved by discussion. All relevant studies were included regardless of their MMAT score.

### Data extraction

Data extraction was completed independently by LN and one of the eight members of the review team, listed above, and was conducted in parallel to the quality assessment. Data items were extracted using a pre-piloted electronic form under the headings listed in Table 2. The two independent data extraction forms for each article were compared and harmonised by MS and CH, who referred back to the original articles where disagreement occurred. The impacts of the clinic academic activity were broadly characterised using the section headings from the VICTOR (making Visible the ImpaCT Of Research) framework [22]. The VICTOR framework was developed for individual research teams to record the impact of their study and is endorsed by the UK National Institute for Health Research [23]. It comprises a series of open ended questions categorised under seven sections: health benefits, safety and quality improvements for research participants and carers during the study; service and workforce impacts; research profile of the organisation and research capacity; economic impacts; organisation’s influence and reputation; knowledge generation and exchange. Following piloting for this systematic review, the headings were modified to include a section for impacts to the individual, and research profile and research capacity and the organisation’s influence and reputation were merged.

**Table 2:** Data extraction items

CHARACTERISTICS OF THE STUDY	CATEGORY OF IMPACT REPORTED
Years of publication and data collection	Impacts to patients (or families/carers)
Location (country and clinical setting)	Changes to service provision
Type of publication	Impacts to research profile of the organisation
Study design	Economic impacts
Clinical background and number of participants	Impacts to staff recruitment or retention
Study aims/objectives	Contribution to knowledge exchange
Nature of the clinical academic activity	Impacts to the individual clinical academic
	Any other impacts

Categories of impact were based on the VICTOR tool [22]

### Data synthesis

Extracted data for each of the pre-identified categories of impact (Table 2) were independently analysed by two members of the review team (LN and one of CH, AA, MS, JC, LSB, MC and LB) to create a thematic synthesis. This involved independently coding the data to identify recurring, unique and contradictory content and using the codes to independently summarise the content of the theme in a series of sub-themes [24]. The findings were discussed and agreed together by the two independent reviewers for each category of impact. The final analysis for all categories of impact was discussed and refined by CMA, MW and LN.

## Results

### Study characteristics

A total of 2704 articles were identified after removal of duplicates, of which 20 met the review eligibility criteria (Fig. 1) [13, 16, 17, 25–41]. The most common reasons for exclusion were that the study population did not involve clinical academics, that is clinicians who were also involved in research activity; or the study assessed the amount of research interest/activity, rather than the impacts of this activity (Additional File 3). Of the included articles, nine reported qualitative data [17, 25, 29–31, 33, 36, 38, 41], three reported quantitative data [28, 35, 37] and eight reported a mixture of both [13, 16, 26, 27, 32, 34, 39, 40]. Sixteen were peer reviewed journal articles and four were organisational reports. Publication dates ranged from 2003 to 2019 and the geographical distribution was: Europe (including the UK) 10; North America 5; Australasia 4; Middle East 1 (Table 3).

**Table 3:** Details of the included studies

AUTHOR, PUBLICATION TYPE AND YEAR	LOCATION (SITE AND COUNTRY)	STUDY DESIGN	CLINICAL DISCIPLINE	NUMBER OF PARTICIPANTS	STUDY/REPORT AIMS	NATURE OF CLINICAL ACADEMIC ACTIVITY
Bäck-Pettersson [25] Journal article 2012	Healthcare organisations & universities, Western Sweden	Qualitative: focus groups	Nursing	12	Describe clinical nurses’ experiences of participating in a Research and Development Programme and the influence on research interest and ability to conduct and apply nursing research	*Name:* Research and Development Programme *Duration:* 2 years *Research:* Conducted and presented a research project from idea to publication *Training:* Masters degree
Higgins [30] Journal article 2010	Acute care & community settings, Australia	Qualitative: nominal group technique	Nursing	Not reported	Explore experiences of nurses who have undertaken clinically based research and document the issues, challenges and benefits	*Name:* Clinicians, academics and researchers *Duration:* Not applicable *Research:* Not reported *Training:* Not reported
Kluijtmans [31] Journal article 2017	Single university, Netherlands	Qualitative: semi-structured interviews	Nursing, physiotherapy	14	Explore how recent nurse- and physiotherapist-scientists perceive their professional identities and experience the crossing of boundaries between care and research	*Name:* Clinician-Scientist Programme *Duration:* 3 years, 20 h/week *Research:* Not reported *Training:* 1-year pre-masters and 2-year masters programmes
Siedlecki [38] Journal article 2016	Large healthcare system, Midwest USA	Qualitative: semi-structured interviews	Nursing	26	Develop a theoretical understanding of the conduct of research by clinical nurses	*Name:* Research-active nurses *Duration:* Not applicable *Research:* Principal investigator for clinical nursing research study (not in fulfilment of educational training) *Training:* Not reported
Wenke [41] Journal article 2017	State healthcare organisation, Victoria, Australia	Qualitative research: semi-structured interviews & focus group	Allied health professions	Interviews: 8 research practitioners and 8 line managers Focus groups: 28	Identify and explore the impact of funded research positions on building allied health research capacity within the organisation. Describe the mechanisms that enable and/or hinder the impact of the research positions in building allied health research capacity	*Name:* Allied health research practitioners (postdoctoral) *Duration:* Not applicable *Research:* Provided research support and conduct their own research *Training:* Provide research supervision and training for allied health professionals
Brooks Carthon [33] Journal article 2017	Single healthcare organisation & university, USA	Unclear – descriptive case study	Nursing	2	Overview and description of a Research Scholars Programme, including: design, conceptual framework, resource requirements and effect on institutional partners and participants	*Name:* Research Scholars Programme *Duration:* 1.5-years, 16 h/month *Research:* Involved with existing research team *Training*: Delivered by postdoctoral researcher
Association of UK University Hospitals [17] Report 2016	National university hospitals, UK	Unclear – descriptive case studies	Nursing, midwifery, allied health professions	32	Provide healthcare providers with practical advice to develop and sustain clinical academic roles with illustrative case studies	*Name:* Clinical academic roles *Duration:* Mixed *Research:* Clinically focused *Training:* pre-doctoral, doctoral and postdoctoral schemes
Department of Health and Social Care [29] Report 2012	Department of health and Social Care, UK	Unclear – descriptive case studies	Nursing, midwifery, physiotherapy	3	Provide support for a strategy to develop the role of clinical academic researchers within nursing, midwifery and allied health professions	*Name:* Clinical Academic Training Programmes *Duration:* Mixed *Research:* Clinically focused *Training:* pre-doctoral, doctoral and postdoctoral schemes
Nursing, Midwifery and Allied Health Professions Research Unit [36] Report, 2017	Healthcare organisations & universities, Scotland	Unclear –descriptive case studies	Nursing, midwifery and allied health professions	11	Showcase the benefits to health and social care of adopting a clinical academic approach	*Name:* Clinical academic approach *Duration:* Mixed *Research:* Clinically focused *Training:* pre-doctoral, doctoral and postdoctoral schemes
Chan [28] Journal article 2010	Cancer care, tertiary hospital, Australia	Unclear – quantitative case study	Nursing	1	Review the design, implementation and evaluation of a Nurse Researcher Project led by an advanced practice level nurse researcher	*Name:* Nurse Research Project *Duration:* *Research:* Development, coordination, implementation and evaluation of nursing research projects *Training:* Not reported
Nazer [35] Journal article 2017	University hospital, Jordan	Quantitative service evaluation	Pharmacy	13	Describe the development of a structured Research Training Programme and evaluate the number of departmental research projects and publications. Data collection over 5 years	*Name:* Pharmacy Research Training Programme *Duration:* Not reported *Research:* Conducted individual research projects *Training:* Tailored education sessions and assignments
Pomeroy [37] Journal article 2003	Research & Development Directorate, North West England, UK	Quantitative survey	Nursing, midwifery, allied health professions	18	Describe the structure and process of a ‘hands-on’ Clinical Research Secondment scheme with the Stroke Associate Therapy Research Unit	*Name:* Research Secondment *Duration:* 1 year (part time) *Research:* Involved in existing projects withing the research unit Training: Research learning programme
Black [26] Journal article 2019	Academic health science organisation, Western Canada	Mixed methods: survey & semi-structured interviews	Nursing and other disciplines (e.g. dietetics, pharmacy, social work)	Survey: 31 Interviews: 11	Review of Research Training Programme after 5 years, including: extent of changes to practice; impact on evidence-based practice; interest in advance education; research engagement; dissemination activities	*Name:* Research Training Programme *Duration:* Not reported *Research*: Conducted research project in own clinical setting (funding provided) *Training:* Research workshops
Bramley [27] Journal article 2018	University hospital, UK	Mixed methods: survey, case studies & quantitative service evaluation	Nursing	7	Pilot the Chief Nurse Excellence in Care Junior Fellowship, document process of implementation and capture outcomes in relation to fellows, patients and the wider organisation	*Name:* Chief Nurse Excellence in Care Junior Fellowship *Duration:* 1 (part time) *Research:* Undertook quality/practice improvement activity within clinical area *Training:* Bespoke training
Hiley [16] Report 2018	Health Education England, West Midlands, UK	Mixed methods: survey & semi-structed interviews	Nursing, midwifery, allied health profession, healthcare scientists, pharmacists	Survey: 53 Interviews: 25	Understand the value of Clinical Academic Programmes for participants and employing healthcare organisations. To determine the barriers and enablers to continuing a clinical academic career	*Name:* Clinical Academic Internship Programme and Masters to Doctorate Bridging Programme *Duration:* 6 to 9-months (part time) *Research:* Completed research placements *Training:* Bespoke training to develop clinical academic portfolio
Leung [32] Journal article 2012	University hospitals, Toronto, Canada	Mixed methods: survey & quantitative service evaluation	Nursing	9	Report development, delivery and evaluation of a research training and mentorship programme	*Name:* Oncology/Supportive Care Research Mentorship Programme *Duration:* 9 months (part time) *Research:* Conducted research and/or disseminated findings with academic and mentorship support *Training:* Research training sessions
McKee [34] Journal article 2017	Acute hospital, Ireland	Mixed methods: survey, quantitative service evaluation & focus groups	Nursing	7	Describe and evaluate the creation of small research groups for nurses supported by academics and research fellows, and aimed at increasing research participation in advanced clinical nursing roles	*Name:* The intervention *Duration:* 1-year *Research:* Individual research projects *Training:* Experiential learning, research methodology and bespoke training
Trusson [39] Journal article 2019	Healthcare organisations, East Midlands, UK	Mixed methods: survey & in-depth interview	Nursing, midwives, allied health professions	Survey: 67 Interviews: 16	Track progression of clinical academics to explore challenges in combining academic study with clinical practice, and to demonstrate impact on patient outcomes	*Name:* Clinical academic Careers *Duration:* Mixed *Research:* Clinically focused *Training:* East Midlands Clinical Academic practitioner network
Turkel [40] Journal article 2008	Community hospital, USA	Mixed methods: survey & appreciative inquiry	Nursing	7	Develop and run a Research Fellowship Programme. Measure impacts on the participants; present the research projects and evaluate their impacts; and present the financial costs of the programme	*Name:* Nursing Research Fellowship *Duration:* 1 year (part time) *Research:* developed research proposal, conducted the study, disseminated findings *Training:* Structured educational programme and mentoring
Wenke [13] Journal article 2018	Healthcare organisation, Queensland, Australia	Mixed methods: longitudinal survey, quantitative & qualitative aspects	Allied health professions	16	Evaluation of a short-term Research Funding Initiative on clinician research capacity, research output and satisfaction	*Name:* Research Funding Initiative *Duration:* 6 months (part time) *Research:* Varied activities including ethics applications, data collection, data analysis, systematic review, writing for publication *Training:* Mentorship and support from the Allied Health Research Fellow

### Participants

A variety of healthcare professions outside medicine were included. Eight articles involved mixed professional groups, most commonly nursing, midwifery and one or more of the allied health professions. Nine articles were specific to nursing, two to allied health professions and one to pharmacy. The nature of the clinical academic activity was not consistent. All articles discussed clinicians conducting research in clinical practice, however some also incorporated formal educational components at masters or doctoral level and others involved short programmes of research training and/or mentorship (Table 3).

### Methodological assessment

**Fig. 2:**
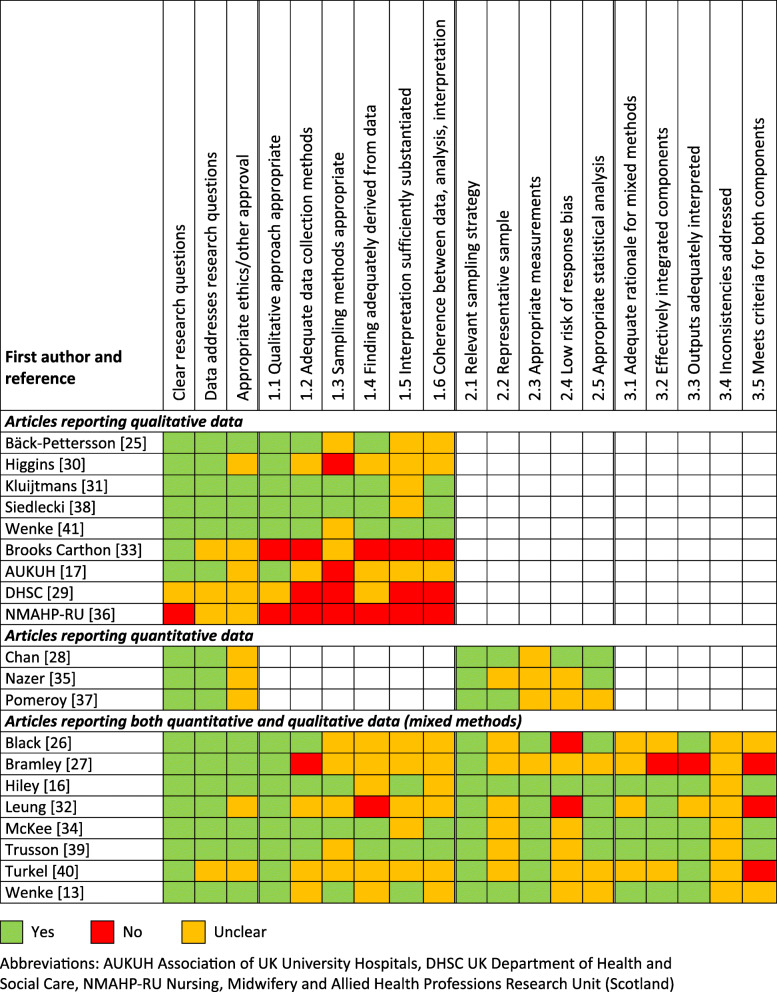
Quality assessment scores for included articles using the Mixed Methods Assessment Tool

Outcomes of the MMAT assessment [20] are shown in Fig. 2 and are available in full via the Open Science Framework [18]. No articles met all quality assessment criteria, although three qualitative studies were rated as having a single area of concern [31, 38, 41]. Common issues with study quality and risk of bias for qualitative and mixed methods studies were a lack of clarity in how the findings were derived from the data and a lack of coherence between data, analysis and interpretation. Common issues with quantitative and mixed methods studies were a lack of information about the measurement tools/methods and a lack of consideration of response bias. Common issues across all study types were inadequate sampling methods and a lack of reporting of ethics/other approvals.

### Reported impacts of clinical academic activity

Reported impacts were categorised into seven main themes based on the modified section headings for the VICTOR framework (Table 2). The distribution of the reported impact themes across the articles included in this review are presented in Table 4. Extracted and coded data for each theme was used to generate the sub-themes, which are described below and presented with illustrative excerpts from the included articles. The full framework of themes, sub-themes and additional quotes is provided in Additional File 4. Most of the extracted impacts were positive in nature, reflecting the aims of the included papers (Table 3), however challenges for the individual clinical academics and their healthcare teams were also described.

**Table 4:** Themes of impact reported in the included papers

	PATIENTS	SERVICE PROVISION		RESEARCH PROFILE, CULTURE & CAPACITY			ECONOMIC	RECRUITMENT & RETENTION	KNOWLEDGE EXCHANGE	INDIVIDUAL
		***Clinical service***	***Clinical academic workforce***	***Profile***	***Culture & capacity***	***Both***				
Bäck-Pettersson [25]	✓	✓	✓	✓	✓	✓	✓	✓	✓	✓
Higgins [30]	✓	✓	✓	–	✓	✓	✓	–	✓	✓
Kluijtmans [31]	✓	✓	✓	–	✓	–	✓	✓	✓	✓
Siedlecki [38]	✓	✓	–	–	✓	✓	–	–	✓	✓
Wenke [41]	✓	✓	✓	✓	✓	✓	✓	✓	✓	✓
Brooks Carthon [33]	✓	✓	✓	–	✓	✓	✓	✓	✓	✓
AUHUK [17]	✓	✓	✓	✓	✓	✓	✓	✓	✓	✓
DHSC [29]	✓	✓	✓	✓	✓	✓	–	–	✓	✓
NMAHP-RU [36]	✓	✓	✓	–	✓	✓	✓	✓	✓	✓
Chan [28]	✓	–	✓	✓	✓	–	✓	–	✓	✓
Nazer [35]	–	–	✓	✓	✓	–	✓	–	✓	✓
Pomeroy [37]	–	✓	✓	✓	✓	✓	✓	✓	✓	✓
Black [26]	✓	✓	–	✓	✓	✓	–	–	✓	✓
Bramley [27]	✓	✓	✓	✓	✓	✓	✓	✓	✓	✓
Hiley [16]	✓	✓	✓	✓	✓	✓	✓	✓	✓	✓
Leung [32]	–	✓	✓	✓	✓	–	–	–	✓	✓
McKee [34]	✓	✓	✓	✓	✓	✓	✓	–	✓	✓
Trusson [39]	✓	✓	✓	–	✓	✓	✓	✓	✓	✓
Turkel [40]	✓	✓	✓	–	✓	✓	✓	✓	✓	✓
Wenke [13]	–	✓	✓	–	✓	✓	✓	✓	✓	✓

*Abbreviations*: *AUKUH* Association of UK University Hospitals, *DHSC* UK Department of Health and Social Care, *NMAHP-RU* Nursing, Midwifery and Allied Health Professions Research Unit (Scotland)

#### Impacts for patients

The reported impacts for patients focused on beneficial *changes to service provision* that arose as a result of local clinical academic activity, and wider *access to evidence-based healthcare* as a result of the promotion of evidence-based practice across research-active teams and departments:

*“This project demonstrated significant improvements in the neutropenic patient pathway, enhancing experience and outcomes for patients and a reduction in unnecessary admissions.”* [27]

*“Behaviours learned during the programmes provided benefits for improving the quality of care delivered within services … Respondents reported they discussed evidence with colleagues, searched the literature for evidence updates, questioned and used evidence to inform their practice following completion of the programme.”* [16]

It was suggested that these practice-changes were associated with *improved patient/carer experiences,* particularly because clinical academic research was directed at issues that were *meaningful to patients* and focused on patient experience in addition to clinical outcomes:

*“My research has demonstrated the benefits of these kinds of approaches both to patients’ quality of life, and patients, carers, friends and families’ experience of palliative care services.”* [36]

*"[It is a] synergistic relationship, as research knowledge improves the care I provide, but the close patient contact allows me to identify areas that require further research."* [29]

Furthermore, clinical academics reported a drive to challenge and *improve their own clinical practice and that of their team,* with the goal of improving all aspects of patient care:

*“Participants expressed how their involvement in the innovation increased their observation of their own clinical practice, brought the research back to practice, enhanced practice development and the clinical role overall while contributing to improved patient care.”* [34]

#### Impacts on service provision and workforce

##### i. Clinical service provision

In addition to the identified impacts to patients (theme 1), changes in practice as a result of the clinical academic activity were also regarded as beneficial to the clinical service through *improved care delivery and pathways*. This included the introduction of new equipment, better integration of clinical teams, efficiencies, cost-savings and securing new clinical funding:

*“Research positions supported projects that led to changes in service delivery models, with [one] manager commenting, ‘ … it’s amazed me that through the research grant that she got for that project, she has now generated for the Health Service recurrent money for the full time [implementation of the] … rural allied health model’.”* [41]

*“One participant’s intervention removes the need for GPs’ referral for physiotherapy, potentially saving ‘multimillion pounds’ across the NHS [National Health Service], and has subsequently been recognised in the NHS long-term plan.”* [39]

It was reported that clinical academics remained up to date with the relevant literature and clinical guidelines and were able to *translate research into practice and implement the evidence*. They were also able to share these skills with their clinical team to support their colleagues in the delivery of high-quality care:

*“As a clinical academic midwife my aim is to bring more evidence into practice and assist other midwives in doing the same.”* [29]

*“Most of this reported activity focused on reviewing published evidence in relation to clinical practice but participants also reported involvement in facilitating/enhancing research skills in other clinicians.”* [37]

The need to find backfill was reported as a negative consequence of *releasing clinical staff for research*. In some instances, it was not possible to find backfill at the same clinical grade as the clinical academic, potentially leaving a deficit in the clinical service. However, backfill posts were also seen as opportunities for other clinicians to gain experience by acting-up into the role:

*“Release from the workplace, despite the employer grant, was in some cases problematic. Finding appropriately skilled staff to cover services particularly in highly specialised areas and, or recruiting to short term, often part time, vacancies were challenges. In contrast some managers saw this as an opportunity to give other staff the chance to act up, for succession planning, or worked creatively to make release possible.”* [16]

*“Protected time for the APNs [Advanced Practice Nurses] (i.e., at least one day a week) to engage in research activities was crucial to the program and, at times, difficult to achieve.”* [32]

The majority of the described clinical academic activity centred on short-term secondments, which elicited issues with the *return to clinical practice from a research role*. These included difficulties in maintaining evidence-based practice due to time constraints and a lack of opportunity to use the research skills that had been developed:

*“On my return to work I was unable to continue to facilitate evidence-based practice as much as I would have liked due to time constraints. Within my working role there was no dedicated time to devote to evidence-based activity.”* [37]

##### ii. Clinical academic workforce

Clinical academic infrastructure was described in terms of fellowship and career pathways. Steps were taken to ensure visibility of these opportunities to facilitate the development of research capacity. However, it was also noted that the absence of established *clinical academic career structures* resulted in a perceived lack of value of these skills and caused difficulties for clinical managers when trying to plan their service:

*“Clinical and academic mentorship exposed the Chief Nurse Fellows to clinical academic career role models, which in turn raised the profile of this alternative career route.”* [27]

*“There’s a huge untapped workforce … with the right support and time we could be doing things more effectively and more efficiently, but that isn’t necessarily valued in organisations. We’ve got to see this many patients, (we’re) not using our skills of criticality, reflectivity; we’re not going to innovate and change practice.”* [39]

*"Imagine being able to continue my research and tie the results directly to clinical practice. I would like there to be an opportunity of this kind. However, there is a lack of services for nurses with higher academic qualifications who want to develop clinical practice.”* [25]

Challenges encountered in *balancing the clinical and research components of the role* were widely reported. Clinical duties were given priority and some articles reported unclear expectations for the research roles, in contrast to established clinical job descriptions:

*“A culture that prioritises practice in the current context means that the doing of nursing work only is seen as core business. This, together with the need for managers and clinicians to make quick decisions in order to achieve short term goals, operates as a disincentive to rigorous research activity at ward level. Within this context, the expectations for those with a research component in their role, is at times unclear.”* [30]

In addition, some articles recounted strategies that aided the development of research skills and clinical academic roles:

*“Also, I think the option of having 0.5FTE [full-time equivalent) backfill was good, as it allowed greater flexibility for staff who have roles that are difficult to backfill full-time, also I thought it was useful to have more thinking time, and time to access support, get feedback etc. Full-time research can be very intense especially when you are not conditioned for it.”* [13]

Where clinical academics identified access to *resources and support*, this was identified as a positive asset. However, a number of articles recalled issues with a lack of manager awareness and support, and insufficient funding for research activities and computer software:

*“I have a very supportive divisional head nurse and have been appointed into a trailblazer post; we haven’t got anything similar within the organisation. So there’s real potential to forge out innovative ways in which clinical academics can fulfil that remit of working in clinical practice and undertaking research, but also pave the way for others that want to come up.”* [39]

*“Biggest challenge: Getting managers on board, in particular releasing staff to take advantage of internship opportunities offered by HEE [Health Education England] Wessex, and recognising that research is essential to the core business of the Trust.”* [17]

*“It’s not the scheme, but greater staff access to relevant software (such as SPSS) would be useful.”* [13]

#### Impacts to the research profile, culture and capacity

Research profile, culture and capacity were interlinked and several of the reported impacts spanned all aspects of this theme. However, *winning research funding and other awards*, *publication* of journal articles and clinical guidelines, and conference *presentations* were primarily considered as contributing to the organisation’s research profile:

*“Since completing the programme - One Chief Nurse Fellow was the first UK nurse to be recognised by the Daisy Foundation and has received a Daisy Award for Extraordinary Nurses. Others have also received nominations and were shortlisted for national nursing awards. In addition, two of the projects are featured on The Academy of Fabulous NHS Stuff.”* [27]

*“As a result of the research activities, seven manuscripts were submitted and accepted for peer-reviewed publications.”* [28]

The provision of *research training and support*, and organisational-wide *research engagement and participation* were commonly reported as beneficial impacts to the organisations’ research culture and capacity:

*“Improved attitudes towards research were noted by a clinician, ‘ … research isn’t this incredibly difficult thing that only very special people can do. Actually, it’s attainable by many and it was quite inspiring actually … I don’t know that that would have been their view prior to this position developing that profile.”* [41]

*“It’s about allowing people to engage with research and become enthused by it. It’s also about having the right leaders who are able to take the step back and say: ‘This is a good use of your time’. You can find better ways of giving care if you have a culture that values research.”* [36]

There were also aspects where the *lack of research culture* within an organisation was seen as a challenge for research involvement:

*“There is the perception that doing research is an ‘imposition, on clinical nursing staff, that it is not ‘real nursing work’; rather, it is a ‘luxury’. When invited by nurse researchers to participate in research activities, clinical nurses often say ‘I don’t have time for this’ and the general attitude is ‘we’ll think about it’, ‘if I have time’, or ‘tell us about the result’. Indeed, in some instances there is a perception amongst discussion group members that projects are undermined through gate-keeping behaviour and lack of support by the ‘sceptics’.”* [30]

Clinical academics were able to promote evidence-based practice among their peers, for example by sharing resources and setting up journal clubs or other special interest groups. This facilitated a shift towards *research becoming embedded in practice*:

*“I now exhort other colleagues to question day-to-day practice and we have introduced a journal club.”* [29]

*“The group process allowed scholars to participate in joint problem solving and enhanced their ability to apply current research to questions arising from clinical practice. The scholars were expected to serve as clinical resources to others in the healthcare system.”* [33]

Three additional elements were identified that encompassed both research profile and research culture/capacity. These were: *building local and external collaborations*, creating *visible clinical academic opportunities* and being seen as *an attractive place to work*:

*“We have increasing numbers of staff involved in research activity, studying for MRes and PhDs, and their research is closely related to their professional practice and aims to improve care. We have five research themes with [the healthcare organisation’s] nurses/midwives leading these and staff linking into these themes for their masters or doctoral study and we are starting to build groups of staff at different points on a clinical academic career pathway. Many staff present their research nationally and internationally and publish widely and some are part of national expert groups, linked to their research.”* [17]

*“People can choose where they want to work. They’ll be looking for organisations that are aspirational. So actually offering innovative career pathways that can intellectually challenge, but also have that direct patient care element, is going to be attractive to a lot of people.”* [39]

#### Economic impacts

The *funding required to support the clinical academic activity* was generally sourced from outside the clinical organisation. Reported benefits of receiving research funding included dedicated time for research training and activity and bringing in additional money to the clinical service:

*“Increased grant income – the value of successful non-medic research grants in 2014–2015 (the last financial year the outcome of all grant applications is known) was £923,495. Appointments of clinical academic posts were achieved by securing external grant funding, use of research capability funding to pump prime, and commitment to 50:50 funding from academic partners.”* [17]

However, there were also issues where funding for research was not available, or was *repurposed from clinical budgets*:

*“Management are more than happy to support research initiatives in principle, however, [they are] usually unable to provide [this] support as they have extremely tight budgets and other clinical management demands.”* [30]

*“The initiative was resourced by the reallocation of nursing/ODP vacancy funding within each clinical division.”* [27]

As discussed in theme 2 (provision of clinical service), it was proposed that clinical academic activity was associated with financial gains in terms of *cost-savings and efficiencies*, although the difficulty of capturing this data was recognised:

*“The amount of money saved by using the scanner and avoiding catheterisation was estimated to be around £1.2m per year. This did not include the cost of bacteraemia attributed to urinary tract infections. Savings associated with using a scanner, such as fewer treatment delays and overnight stays in hospital, were recognised as additional savings. The set up and running costs of a scanner were estimated to be met within six months to two years, after which significant ongoing cost efficiencies would be realised over its eight to ten year lifespan.”* [17]

*“The work had led to a decrease in patients’ clinical stay following surgery from six to four days, resulting in savings that allowed additional needs and demands to be met. We were very open and transparent with the data, and clinical practice changed. We calculated that there was a total of 28,000 bed-days saved per year as a result of this work.”* [36]

*Financial implications for the clinical academics* were also reported. These were largely negative and included reductions in salary and pension contributions, and the need to self-fund conference attendance:

*“Well one of the big decisions I had to make about whether or not to accept the role (associated with a research training award) was the hours and the money because it’s moving to full time, which is fine... Therefore, by moving to full time but losing my enhancements I’ll be on around the same as I get on a good month when I have done lots of nights and weekends. But by working as I’m doing there won’t be any opportunity to do extra shifts, any overtime. So a lot of it was money.”* [16]

*“The presenters had to either self-fund their travel and conference registration, or apply for travel scholarships through internal or external opportunities.”* [28]

#### Impacts on staff recruitment and retention

The lack of clinical academic career opportunities was noted as a challenge that individuals wishing to *maintain a dual role* needed to negotiate. This related to the sub-theme ‘balancing clinical and academic components of the role’ discussed in theme 2 (impacts for the clinical academic workforce), and was identified as a potential driver for individuals to return to full-time clinical work, or move into purely academic roles after completion of their clinical academic activity:

*“The organisational system is perceived as unfamiliar with, and unsupportive of, non-physician clinician scientist positions, and, in consequence, active job crafting is necessary to obtain positions in which such individuals can exert both roles. Dual positions are often a personal combination of jobs instead of being offered from within one institution.”* [31]

*"Unfortunately, I wasn’t offered the chance to implement the results of my study in my organisation, due to the lack of development positions. So, as a result, I have applied for, and been given, a position as a teacher at the university college."* [25]

A particular challenge for healthcare managers was the need to provide backfill or make other arrangements to enable the release of clinical staff for research activities. The need to recruit to backfill posts was discussed in theme 2 (impact to clinical service provision), specifically the sub-themes: release of clinical staff for research and return to clinical practice from a research role.

Several articles reported strategies to *support clinical academics* and increase awareness and access to clinical research opportunities. Where successful, it was suggested that these aided the retention and career progress of staff who were involved in clinical academic activity. Such strategies also contributed to job satisfaction and recruitment more generally and were closely linked with the impacts to the organisation’s research profile, research culture and capacity (theme 3):

*“Since completing their year as a fellow, the entire pilot cohort still works within the organisation, with five of them having moved into junior leadership positions. Although we cannot assume that this would not have been their career trajectory had they not undertaken this fellowship, the skills developed and demonstrated through the initiative are essential for the job specifications of more senior posts.”* [27]

*“Interviewees reported that their department was seen as a more ‘attractive employer’ and was ‘attracting higher calibre staff’. Clinicians described staying in the health service to undertake research, ‘because these opportunities do exist, these really fabulous clinicians that we have just might stay’.”* [41]

#### Impacts to knowledge exchange

Contributions to knowledge transfer were reported in all articles, and there was a large overlap with theme 3 (research profile, culture and capacity). Knowledge exchange activities included formal *dissemination*, such as conference presentations and posters, publications, being an invited speaker and winning prizes and awards that further highlighted the value of the work. It was recognised that there would be a delay between completion of the research activity and delivery of these research outputs:

*“Interviewees provided numerous and diverse examples of presentations at grand rounds, poster presentations and oral presentations at both local and international conferences, including one interviewee who noted her team had presented their research project findings at three international and three local conferences. Another interviewee stated, ‘We have presented at a couple of conferences and we presented at a … convention or meeting and we actually got an award for our poster’.”* [26]

*“Individuals need time in the role as well to get some momentum, get the relationships in the department, get the research programs going and there's usually a delay until you start to see the pure research outputs.”* [41]

Clinical academics also played a role in *developing networks and collaborations* aiding the transfer of knowledge among clinical and academic communities and patient populations. This included sharing their expertise with clinical colleagues and other *practice improvement* strategies aimed at the implementation of clinical guidelines and delivery of evidence-based practice. Again there was a large overlap with theme 2 (impacts to clinical service provision), specifically the sub-theme translation of research into practice and evidence implementation:

*“What I notice clearly is that I’m very well informed about scientific evidence and sharing this information with my colleagues. [I ask them] did you read this? And [I] pass on knowledge in that way.”* [31]

*“After being able to demonstrate the success of the approach locally, David was asked to help with a national roll out, organising with colleagues an audit of all 22 orthopaedic units across Scotland over 12 weeks.”* [36]

#### Impacts to the clinical academic

Many of the themes and sub-themes of impact discussed above had also had a direct influence on the individuals involved in clinical academic activities. Developing networks and collaborations, discussed in theme 6 (knowledge exchange), and building local and external collaborations, discussed in theme 3 (research profile, culture and capacity), were similarly interpreted as individual clinical academics *developing their networks and influence*:

*“I think the impact of the role on me has been quite incredible. … how much you learn about the different disciplines and then develop those networks … it’s been a huge learning curve.”* [41]

Furthermore, clinical academics reported a change in their *attitude to clinical practice,* with greater reflection and questioning of established practice, which was also reflected in theme 1 (impacts for patients), particularly in terms of improved clinical practice and access to evidence-based healthcare:

*“They felt that they had developed from “doers” to “thinkers”, in that they felt more aware of and reflective in relation to their colleagues … The nurses perceived progress in acquiring new knowledge, in spite of language barriers, and recognised the value of scientific knowledge for clinical practice. They experienced healthcare in a ‘new light’ through their knowledge development.”* [25]

The *development of research and leadership skills* was identified as a beneficial effect of being involved in clinical academic activity. In some articles, it was suggested that this unlocked new *career opportunities* for the individuals involved, however in many instances there were no existing roles within the organisation for the research-active clinicians to aspire to, as discussed in theme 5 (recruitment and retention):

*“The majority agreed or strongly agreed that they felt more confident developing a research question (94%/49), searching (87%/45) and appraising (90%/47) literature, challenging practice using evidence (85%/44), assisting others to use critical appraisal skills (79%/41) and engage in the clinical academic training pathway (87%/45).”* [16]

*“Despite their achievements during the PhD, many participants expressed anxieties about their future careers, having been made to move aside clinically in order to progress their academic ambitions, rather than being able to develop their academic and clinical skills in tandem. For example a dietician said: Recently I’ve had to step out of my area of expertise … I’m just doing general, allergies, weight management, which is not my area, but I need to pay the mortgage.”* [39]

Clinical academics appeared to find *self-fulfilment* with their roles, and described a passion for their contributions to clinical research:

*“It is actually exciting to learn that the world does not work the way you thought it did”. 70* [38].

*“I want to do this for me, but I also want to do it for my daughters to show that women can be in science and can lead in these fields and yes we might have to juggle family things and children, but you can do it.”* [39]

*“What accomplishments are you most proud of? Knowing that I am now a subject expert – I get phone calls asking, `How would you handle this?’.”* [40]

The *challenges and sacrifices* of clinical academic roles were widely recounted, examples of which have been included in the previous themes. Financial implications were discussed in theme 4 (economic impacts, sub-theme financial implications for the clinical academic) and the challenge of combining clinical and academic work and identities were included in theme 2 (impacts for the clinical academic workforce, sub-theme balancing clinical and academic components of the role) and theme 5 (impacts on staff recruitment and retention, sub-theme maintaining a dual role). A lack of time for clinical academic activity was another widely reported issue:

*“When you are really interested in something or passionate about it, you use whatever time you have, even if it means writing your proposal after your regular hours at home.”* [38]

*“The time taken to do research is often underestimated and considerable time and effort is often put into preparing a grant application which ultimately may not be successful. Focusing on meeting deadlines and the progress of a project means that less attention can be given to other aspects of work roles. Ultimately, doing research without adequate support or funding becomes a constant juggle.”* [30]

Finally, a few articles reported reflections on *what it takes to be a clinical academic*. These included the qualities of determination, tenacity and resilience, and serendipity:

*“The demanding expectations surrounding a clinical academic role were described by interviewees (participants and managers) and the characteristics and behaviours that were perceived as required for success. These included confidence, doggedness and resilience, reflective skills, criticality, and growing political know-how to better navigate organisations.”* [16]

*“Mentorship (from nursing, midwifery and medical colleagues), determination, tenacity, resilience and serendipity have been key factors in achieving success.”* [17]

## Discussion

This systematic review identified 20 articles that discussed elements of the impact of clinical academic activity among healthcare professionals outside medicine. With the addition of a theme for the impacts to the clinical academic, all reported types of impact could be mapped to the VICTOR framework creating the following themes: impacts for patients; impacts for service provision and workforce; impacts to research profile, culture and capacity; economic impacts; impacts on staff recruitment and retention; impacts to knowledge exchange; and impacts to the clinical academic. In order to develop and evaluate clinical academic roles for healthcare professionals outside medicine, the range of impacts of this clinical academic activity need to be understood and valued by healthcare leaders and managers. This review has systematically identified and mapped the nature of the impacts reported in the literature, and forms a valuable resource for healthcare services looking to develop and evaluate these roles at local and national levels.

Within the main headings of impact described above, we identified several similar sub-themes that cut across the different categories of impact. Sub-themes described the content of each of the categories of impact and included perceived enablers of creating the desired impact and associated detrimental features. Notably, the sub-themes that reflected the challenge of maintaining or balancing the clinical and academic components of the role contributed to four main themes. Within clinical service provision (theme 2i), this related to the need for clinical services to manage both the release of clinical staff for research, and their return to clinical practice after research secondments. For the clinical academic workforce (theme 2ii), this led to individuals and team members being required to adapt to the different pace and duties associated with research and clinical work. The process of showcasing a visible clinical academic pathway (that incorporated both research and clinical activities) was identified as key feature of building research profile, culture and capacity (theme 3), and being able to offer suitable clinical academic posts was important for staff recruitment and retention (theme 5). Finally, being able to work and develop in both clinical and academic roles offered self-fulfilment for the individual clinical academic (theme 7). Similarly, the creation and implementation of new evidence was also a component of several themes, as was the development of collaborations and networks.

The multifaceted nature of research impact identified in this review illustrates that different aspects of clinical academic activity may be perceived as having both positive and negative impacts. Furthermore, these conflicting impacts may apply to the same individual or across different stakeholders. Different aspects of research impact may be more or less important in different contexts and the relative value of these different impacts will need to be considered to enable meaningful evaluation [42–44].

This systematic review was deliberately broad in scope to allow the identification of the whole range of impacts associated with clinical academic activity. The lack of an agreed and consistently used definition of clinical academic proves problematic and has been discussed elsewhere [45, 46]. Clinical academic activity (defined here as the involvement of practising clinicians in research) in the included articles incorporated a range of research fellowships and research training programmes, and/or in-practice mentorship and research support. The aims of the articles varied. Many described and evaluated specific interventions that were aimed at increasing research activity among healthcare professional group(s), while others provided summary case studies of individuals who had been successful in a clinical academic role.

The inclusion of grey literature increased the breadth of the review, particularly given the finding that the impacts of non-medical healthcare research are underrepresented in the academic literature [47]. However, it is acknowledged that the methodological quality of the included institutional reports was lower than the standards for peer-reviewed publication. Data obtained from the institutional reports were largely positive reflections of strategies that had been put into place to encourage and support research activity among healthcare professionals outside medicine. Expressions of the less positive aspects of these strategies may therefore have been excluded or not collected by the authors. Importantly, the distribution of the seven identified themes of impacts did not differ between organisational reports and peer review journals, illustrating that the types of impact that were considered important by study participants and organisations were similar.

Existing reviews on the impact of clinical academic activity have focused on individual clinical groups within the non-medical workforce [10] or at the level of the healthcare institution [1]. Boaz et al. found that healthcare organisations which deliberately integrated research into their practice and fostered research engagement reported improved healthcare performance including clinical outcomes and processes of care, and our review also identified similar improvements. However, Boaz et al.’s review included papers focused primarily on research led by clinical academic doctors, and the impacts of the research processes on the clinical teams and the individuals involved was not reported [1]. Wenke and Mickan identified four themes of impact associated with allied health research positions based in clinical settings: increased individual research skills and participation; increased research activity; improved research culture and attitudes; and increased team and organisational level skills [10]. These features were also described within the current review under the themes: impact for patients; impacts to the clinical academic; impacts to research profile, culture and capacity; and impacts for service provision and workforce. In addition, we also identified impacts on staff recruitment and retention, knowledge exchange and economic impacts.

The distribution of impacts reported in the current review did not differ in relation to the clinical groups involved in each of the included articles, indicating that similar methods of capturing the impact of clinical academic research activity could be applied across the professions outside medicine, rather than being discipline-specific. A similar systematic review search strategy could also be applied to assess the reported impact of clinical academic activity by alternative and complementary therapy practitioners.

The impacts reported in the included articles were largely qualitative reports. Some studies incorporated quantitative data capture tools, such as the Research Capacity and Culture tool [48, 49] and the WReN (Wessex Research Network) spider [50, 51], or counts of publications, presentations and awards. The quantitative measures appeared to explore a discrete component of research impact, whereas the qualitative data provided a broad picture of the impacts in different contexts and uncovered both intended and unintended consequences of the research activity. Reed et al. proposed five impact evaluation typologies (experimental and statistical methods, systems analysis methods, textual, oral and arts-based methods, indicator-based approaches, and evidence synthesis approaches); our findings illustrate the first and third of these categories [44]. Future work should explore standardised methods of capturing the research impact that address the full range of impacts identified in this systematic review and are specific to the context of clinical academics outside medicine. With the desire for at least 1% of the UK NMAHP workforce to be clinical academics by 2030 [8], policy makers will need to consider, promote or potentially mitigate the different types of impacts that this systematic review identified in connection with these roles.

The VICTOR framework [22] was used to guide data extraction in the current review, with a priori modifications to include the impacts to the individual clinical academic and to merge the category relating to the organisation’s influence and reputation with the category for research profile, culture and capacity. No further refinements were made during the analysis process as all reported impacts were able to be mapped. While other research impact frameworks exist [14, 44], our findings suggest that the VICTOR tool may be a good starting point for capturing the nature of research impact that is important for clinical academic healthcare research outside the medical professions, and it is already endorsed for use in the UK [23]. The identification of sub-themes that crossed one or more of the main impact themes indicate that these may be key areas to explore, particularly for organisations looking to support and increase academic activity among these clinical groups.

### Limitations

The systematic review team comprised research-active clinicians from professions outside medicine, and therefore the review took place through this lens. Steps were taken to facilitate objectivity, including: a clearly defined protocol [18]; two or more reviewers independently conducting each stage of the review; inclusion of reviewers with different levels of clinical and research experience; and the provision of oversight by senior (clinically-active) academics. We acknowledge that the thematic analysis and coding of the extracted data may have been interpreted differently by reviewers from different backgrounds. Furthermore, the definition of clinical academic activity used in our review differs from that used elsewhere [45, 52]. However, the absence of an agreed definition has been recognised [45] and the overlap of our findings with the existing literature support our review processes and findings [1, 10].

As with other reviews of research impact, there is a risk that relevant studies were excluded due to poor indexing in the medical databases [14]. We took the additional step of including grey literature searches in both established repositories and through a naïve web search engine in an attempt to maximise the identification of eligible articles, but accept that articles may still have been missed if they were not identified through these mechanisms. We did not formally screen the reference lists of the included articles, and accept that this may have yielded additional studies.

The MMAT tool used for methodological assessment of the included articles was designed for the appraisal of mixed methods studies [20], although our review included mixed methods, qualitative, and quantitative articles. The MMAT was chosen to allow the same criteria to be applied across all included articles using the relevant sub-sections as appropriate. Quality assessment scores were not used to determine how the extracted data was incorporated into the thematic synthesis. We acknowledge that the presented synthesis therefore includes the findings from studies and organisational reports across the spectrum of methodological quality. However, no theme or sub-theme solely comprised data from articles that were assessed to be of lower quality.

The application of alternative impact frameworks would have yielded different theme headings, as these were taken directly from the VICTOR terminology. However, the use of an established impact assessment tool aided transparency and consistency of data extraction and categorisation. The coding and resulting theme descriptions were created through an inductive process that explored the meaning of the extracted data, rather than looking to specifically fit it to the VICTOR headings. The descriptions of the content of each theme illustrate the available data.

Finally, the aim of our review was to capture the range of impacts associated with clinical academic activity outside medicine. We have highlighted the key themes of impact and described the characteristic content of these themes. While this work contributes to the existing discussion around research impact, it does not explore the utility of capturing and comparing the reported impacts using a standardised method in a clinical research setting.

## Conclusion

Twenty articles were identified that reported the impact of clinical academic activity among the healthcare professions outside medicine. These impacts could be mapped using a modified VICTOR framework and were classified as: impacts for patients; impacts for service provision and workforce; impacts to research profile, culture and capacity; economic impacts; impacts on staff recruitment and retention; impacts to knowledge exchange; and impacts to the clinical academic. With our addition of impacts to clinical academics, the VICTOR tool may be a useful starting point for individuals and organisations to record the impact of their research activity, although further work is needed to establish its utility. This review identified several sub-themes of impact that crossed one or more of the main themes: the challenges and benefits of balancing clinical and academic roles; the creation and implementation of new research evidence; and the development of collaborations and networks. These are likely to be key areas for organisations to explore when looking to support and increase academic activity among healthcare professionals outside medicine.

## Supplementary Information

**Additional file 1.** Example search strategy for Medline. Search strategy terms.

**Additional file 2.** Mixed Methods Appraisal Tool. Quality appraisal form and instruction for use.

**Additional file 3.** Articles excluded during full text screen and reasons for exclusion. References of the excluded articles and reason for exclusion.

**Additional file 4.** Coding framework for the types of impact identified and exemplar quotes. Full listing of the theme headings with descriptors and supplementary quotes.

## Data Availability

The datasets generated and analysed during the current review are available in the Open Science Framework (OSF) repository (https://osf.io/gj7se). All other relevant data are included as supplementary files.

## Abbreviations

AHP: allied health professional (includes: art therapists; drama therapists; music therapists; chiropodists/podiatrists; dietitians; occupational therapists; operating department practitioners; orthoptists; osteopaths; paramedics; physiotherapists; prosthetists and orthotists; radiographers; and speech and language therapists)
AUKUK: Association of UK University Hospitals
DHSC: Department of Health and Social Care (UK)
MMAT: Mixed Methods Appraisal Tool
NMAHPs: nurses, midwives and allied health professionals
NMAHP-RU: Nursing, Midwifery and Allied Health Professions Research Unit (Scotland)
PRISMA: preferred reporting items for systematic reviews and meta-analyses
VICTOR: making Visible the ImpaCT Of Research framework, a research impact capture tool

## References

[1] Boaz A, Hanney S, Jones T, Soper B (2015). Does the engagement of clinicians and organisations in research improve healthcare performance: a three-stage review. BMJ Open.

[2] Jonker L, Fisher SJ, Dagnan D (2020). Patients admitted to more research-active hospitals have more confidence in staff and are better informed about their condition and medication: results from a retrospective cross-sectional study. J Eval Clin Pract.

[3] Majumdar SR, Roe MT, Peterson ED, Chen AY, Gibler WB, Armstrong PW (2008). Better outcomes for patients treated at hospitals that participate in clinical trials. Arch Intern Med.

[4] Jonker L, Fisher SJ (2018). The correlation between National Health Service trusts’ clinical trial activity and both mortality rates and care quality commission ratings: a retrospective cross-sectional study. Public Health.

[5] Medical Schools Council. Clinical academia. 2018. https://www.medschools.ac.uk/our-work/clinical-academia. Accessed 4 Mar 2020.

[6] Healthcareers NHS. Clinical academic medicine. 2017. https://www.healthcareers.nhs.uk/explore-roles/doctors/career-opportunities-doctors/clinical-academic-medicine. Accessed 1 Oct 2020.

[7] National Institute for Health Research. Building a research career: A guide for aspiring clinical academics (excluding doctors and dentists) and their managers. 2016. https://www.clahrc-eoe.nihr.ac.uk/2016/02/building-a-research-career-a-guide-for-aspiring-clinical-academics-and-their-managers-from-the-nihr/. Accessed 16 Oct 2020.

[8] Westwood G, Richardson A, Latter S, Macleod Clark J, Fader M (2018). Building clinical academic leadership capacity: sustainability through partnership. J Res Nurs.

[9] National Institute for Health Research. HEE-NIHR Integrated Clinical Academic Programme. 2019. https://www.nihr.ac.uk/explore-nihr/academy-programmes/hee-nihr-integrated-clinical-academic-programme.htm. Accessed 4 Mar 2020.

[10] Wenke R, Mickan S (2016). The role and impact of research positions within health care settings in allied health: a systematic review. BMC Health Serv Res.

[11] Hill NL, Yevchak A, Kolanowski AM, Penrod FJ, Milone-Nuzzo PF, Sawyer AM (2014). What it takes: perspectives from developing nurse scientists. J Nurs Educ.

[12] Munro S, Hendrix CC, Cowan LJ, Battaglia C, Wilder VD, Bormann JE, Uphold CR, Sullivan SC (2019). Research productivity following nursing research initiative grants. Nurs Outlook.

[13] Wenke R, Weir KA, Noble C, Mahoney J, Mickan S (2018). Not enough time for research? Use of supported funding to promote allied health research activity. J Multidiscip Healthc.

[14] Cruz Rivera S, Kyte DG, Aiyegbusi OL, Keeley TJ, Calvert MJ (2017). Assessing the impact of healthcare research: a systematic review of methodological frameworks. PLoS Med.

[15] Adam P, Ovseiko PV, Grant J, Graham KEA, Dowd AM, Boukhris OF (2018). ISRIA statement: ten-point guidelines for an effective process of research impact assessment. Heal Res Policy Syst..

[16] Hiley J, Begg C, Swift A, Topping A. West midlands clinical academic careers Programmes for nurses, midwives, allied health professions, pharmacists and healthcare scientists (NMAHPPS). Evaluation Report 2018. https://www.birminghamhealthpartners.co.uk/education/clinical-academic-careers-programmes/. Accessed 6 Mar 2020.

[17] Association of UK University Hospitals. Transforming healthcare through clinical academic roles in nursing, midwifery and allied health professions: A practical resource for healthcare provider organisations. 2016. https://councilofdeans.org.uk/2016/11/transforming-healthcare-through-clinical-academic-roles-in-nursing-midwifery-and-allied-health-professions/. Accessed 18 Oct 2020.

[18] Newington L, Wells M, Alexander CM. What are the impacts of clinical academic activity among practising healthcare professionals outside medicine? A systematic review (protocol). OSF Registry. 2019; https://osf.io/gj7se.

[19] Moher D, Shamseer L, Clarke M, Ghersi D, Liberati A, Petticrew M, et al. Preferred reporting items for systematic review and meta-analysis protocols (PRISMA-P) 2015 statement. Syst Rev. 2015;4. 10.1186/2046-4053-4-1. 10.1186/2046-4053-4-1 PMC4320440 25554246

[20] Hong QN, Pluye P, Fàbregues S, Bartlett G, Boardman F, Cargo M, Dagenais P, Gagnon MP, Griffiths F, Nicolau B, O’Cathain A, Rousseau MC, Vedel I (2019). Improving the content validity of the mixed methods appraisal tool: a modified e-Delphi study. J Clin Epidemiol.

[21] Walsh D, Downe S (2006). Appraising the quality of qualitative research. Midwifery..

[22] Cooke J, Jones N, Holliday J. VICTOR (making visible the ImpaCT of research) pack. A resource for healthcare organisation 2019. https://www.e-repository.clahrc-yh.nihr.ac.uk/visible-impact-of-research/. Accessed 11 Dec 2019.

[23] National Institute for Health Research. A VICTOR-y for measuring research impact in the NHS. 2019. https://www.nihr.ac.uk/blog/a-victor-y-for-measuring-research-impact-in-the-nhs/12081. Accessed 6 Nov 2020.

[24] Thomas J, Harden A (2008). Methods for the thematic synthesis of qualitative research in systematic reviews. BMC Med Res Methodol.

[25] Bäck-Pettersson S, Jensen KP, Kylén S, Sernert N, Hermansson E (2013). Nurses’ experiences of participation in a research and development programme. J Clin Nurs.

[26] Black AT, Ali S, Baumbusch J, McNamee K, Mackay M (2019). Practice-based nursing research: evaluation of clinical and professional impacts from a research training programme. J Clin Nurs.

[27] Bramley L, Manning JC, Cooper J (2018). Engaging and developing front-line clinical nurses to drive care excellence: evaluating the chief nurse excellence in care junior fellowship initiative. J Res Nurs.

[28] Chan R, Gardner G, Webster J, Geary A (2010). Building research capacity in the nursing workforce: the design and evaluation of the nurse researcher role. Aust J Adv Nurs.

[29] Department of Health and Social Care. Developing the Role of the Clinical Academic Researcher in the Nursing, Midwifery and Allied Health Professions. 2012. https://www.gov.uk/government/publications/developing-the-role-of-the-clinical-academic-researcher-in-the-nursing-midwifery-and-allied-health-professions. Accessed 27 Sep 2020.

[30] Higgins I, Parker V, Keatinge D, Giles M, Winskill R, Guest E, Kepreotes E, Phelan C (2010). Doing clinical research: the challenges and benefits. Contemp Nurse.

[31] Kluijtmans M, de Haan E, Akkerman S, van Tartwijk J (2017). Professional identity in clinician-scientists: brokers between care and science. Med Educ.

[32] Leung D, Widger K, Howell D, Nelson S, Molassiotis A (2012). Mentoring advanced practice nurses in research: recommendations from a pilot program. Can Oncol Nurs J.

[33] Brooks Carthon J, Holland S, Gamble K, Rothwell H, Pancir D, Ballinghoff J (2017). Increasing research capacity in a safety net setting through an academic clinical partnership. J Nurs Adm.

[34] McKee G, Codd M, Dempsey O, Gallagher P, Comiskey C (2017). Describing the implementation of an innovative intervention and evaluating its effectiveness in increasing research capacity of advanced clinical nurses: using the consolidated framework for implementation research. BMC Nurs.

[35] Nazer LH, Tuffaha H, Jaddoua S (2017). A program to increase research productivity among hospital pharmacists. J Pharm Pract.

[36] Nursing Midwifery and Allied Health Professsions Research Unit. A clinical academic approach for nurses, midwives and allied health professionals - it’s a no-brainer! 2017. https://www.nmahp-ru.ac.uk/media/microsites/nmahp-ru/documents/Clinical-academic-approach-for-nurses-midwives%2D%2DAHPs.pdf. Accessed 27 Apr 2020.

[37] Pomeroy VM, Tallis RC, Stitt E (2003). Dismantling some barriers to evidenced-based rehabilitation with “hands-on” clinical research secondments. Physiotherapy..

[38] Siedlecki SL, Albert NM (2017). Research-active clinical nurses: against all odds. J Clin Nurs.

[39] Trusson D, Rowley E, Bramley L (2019). A mixed-methods study of challenges and benefits of clinical academic careers for nurses, midwives and allied health professionals. BMJ Open.

[40] Turkel MC, Ferket K, Reidinger G, Beatty DE (2008). Building a nursing research fellowship in a community hospital. Nurs Econ.

[41] Wenke RJ, Ward EC, Hickman I, Hulcombe J, Phillips R, Mickan S (2017). Allied health research positions: a qualitative evaluation of their impact. Heal Res Policy Syst.

[42] Greenhalgh T, Raftery J, Hanney S, Glover M (2016). Research impact: a narrative review. BMC Med.

[43] Bayley JE, Phipps D (2019). Building the concept of research impact literacy. Evid Policy.

[44] Reed MS, Ferré M, Martin-Ortega J, Blanche R, Lawford-Rolfe R, Dallimer M, Holden J (2021). Evaluating impact from research: a methodological framework. Res Policy.

[45] Carrick-Sen D, Moore A, Davidson P, Gendong H, Jackson D (2019). International perspectives of nurses, midwives and allied health professionals clinical academic roles: are we at tipping point?. Int J Pract Learn Heal Soc Care.

[46] Newington L, Alexander CM, Wells M. What is a clinical academic? Qualitative interviews with healthcare managers, research-active nurses and other research-active healthcare professionals outside medicine. J Clin Nurs. 2021;00. 10.1111/jocn.15624. 10.1111/jocn.15624 33370491

[47] Kelly D, Kent B, McMahon A, Taylor J, Traynor M (2016). Impact case studies submitted to REF 2014: the hidden impact of nursing research. J Res Nurs.

[48] Holden L, Pager S, Golenko X, Ware RS (2012). Validation of the research capacity and culture (RCC) tool: measuring RCC at individual, team and organisation levels. Aust J Prim Health.

[49] Matus J, Wenke R, Hughes I, Mickan S (2019). Evaluation of the research capacity and culture of allied health professionals in a large regional public health service. J Multidiscip Healthc.

[50] Luna Puerta L, Apfelbacher C, Smith H (2019). Proliferation of the WReN spider, an instrument to measure health professionals’ experience of research: a bibliographic study. BMC Med Educ.

[51] Smith H, Wright D, Morgan S, Dunleavey J, Moore M (2002). The ‘research spider’: a simple method of assessing research experience. Prim Heal Care Res Dev.

[52] Council of Deans of Health. Nursing, midwifery and allied health clinical academic careers in the UK. 2018. http://councilofdeans.org.uk/wp-content/uploads/2018/08/Nursing-midwifery-and-allied-health-clinical-academic-research-careers-in-the-UK.pdf. Accessed 18 Feb 2020.

