# Supplementary material for: A qualitative systematic review and thematic synthesis exploring the impacts of clinical academic activity by healthcare professionals outside medicine
Source: BMC Health Serv Res. 2021 Apr 29;21:400. doi: 10.1186/s12913-021-06354-y (PMC8082861; doi:10.1186/s12913-021-06354-y)
Supplement: Supplementary file 1 — Additional file 1. Example search strategy for Medline. Search strategy terms. [file 12913_2021_6354_MOESM1_ESM.pdf]

Example search strategy for Medline

POPULATION - Clinical discipline

(Nurs*).ti,ab OR “CLINICAL NURSING RESEARCH”/ OR “NURSING RESEARCH”/
(Midwi*).ti,ab OR MIDWIFERY/

(“Allied health”).ti,ab
(“Allied health profession*”).ti,ab OR “ALLIED HEALTH PERSONNEL”/ OR “ALLIED HEALTH OCCUPATIONS”/

(“Art therap*”).ti,ab OR “ART THERAPY”/
(Dietician*).ti,ab OR (Dietitian*).ti,ab OR (Dietetic*).ti,ab OR (Nutritionist*).ti,ab OR NUTRITIONISTS/
(“Drama therap*”).ti,ab OR (Dramatherap*).ti,ab
(“Music therap*”).ti,ab OR “MUSIC THERAPY”/
(“Occupational therap*”).ti,ab OR “OCCUPATIONAL THERAPISTS”/
(Orthoptist*).ti,ab
(“Operating department practitioner*”).ti.ab OR “OPERATING ROOM TECHNICIANS”/
(Osteopath*).ti,ab
(Podiatr*).ti,ab OR PODIATRY/
(Chiropod*).ti,ab
(Prosthetist*).ti,ab
(Orthotist*).ti,ab
(Paramedic*).ti,ab
(Physiotherap*).ti,ab OR (“Physical Therap*”).ti,ab OR “PHYSICAL THERAPISTS”/
(Radiographer*).ti,ab OR (“Radiation therap*”).ti,ab OR (Radiotherapist*).ti,ab
(“Speech and language therap*”).ti,ab OR (“Speech-language patholog*’).ti,ab OR

(“Clinical psycholog*”).ti,ab
(“Health* scientist*”).ti,ab
(Pharmacist*).ti,ab OR (“pharmacy assistant*”).ti,ab OR (“pharmacy technician*”) OR PHARMACISTS/

AND

INTERVENTION – Clinical academic activity

(“Clinical academic*”).ti,ab
(Clinic* scientist*).ti,ab
(“Clinical investigator*”).ti,ab
(Fellow*).ti,ab
(“Research position*”).ti,ab
(“Research career*”).ti,ab
(“Research role*”).ti,ab
(“Research* training”).ti,ab
(“Research intern*”).ti,ab

AND

OUTCOME – assessment of the clinical academic activity
(Impact*).ti,ab
(Evaluat*).ti,ab
(Assess*).ti,ab
(Framework*).ti,ab
(Culture*).ti,ab
(Capacit*).ti,ab
