# Supplementary material for: A qualitative systematic review and thematic synthesis exploring the impacts of clinical academic activity by healthcare professionals outside medicine
Source: BMC Health Serv Res. 2021 Apr 29;21:400. doi: 10.1186/s12913-021-06354-y (PMC8082861; doi:10.1186/s12913-021-06354-y)
Supplement: Supplementary file 2 — Additional file 2. Mixed Methods Appraisal Tool. Quality appraisal form and instruction for use. [file 12913_2021_6354_MOESM2_ESM.pdf]

Mixed Methods Appraisal Tool (MMAT)

Article number: **Author:** **Reviewer:**

Study design	Methodological quality criteria	Responses			
		Yes	No	Unclear	Comments
Screening (all studies)	S.1. Are there clear research questions?				
	S.2. Do the collected data address these research questions?				
	S.3. Is there appropriate ethics approval?				
Qualitative	1.1. Is the qualitative approach appropriate to answer the research question?				
	1.2. Are the data collection methods adequate to address the research question?				
	1.3. Are the sampling methods and sample appropriate?				
	1.4. Are the findings adequately derived from the data?				
	1.5. Is the interpretation of the results sufficiently substantiated by data?				
	1.6. Is there coherence between the qualitative data sources, collection, analysis and interpretation?				
Quantitative descriptive	2.1. Is the sampling strategy relevant to address the research question?				
	2.2. Is the sample representative of the target population?				
	2.3. Are the measurements appropriate?				
	2.4. Is the risk of nonresponse bias low?				
	2.5. Is the statistical analysis appropriate to answer the research question?				
Mixed methods	3.1. Is there an adequate rationale for using a mixed methods design to address the research question?				
	3.2. Are the different components of the study effectively integrated to answer the research question?				
	3.3. Are the outputs of the integration of qualitative and quantitative components adequately interpreted?				
	3.4. Are divergences and inconsistencies between quantitative and qualitative research adequately addressed?				
	3.5. Do the qualitative and quantitative components of the study meet the quality criteria provided in sections 1-2?				

Explanations

<table>
<tr><th>1. Qualitative studies</th><th>Methodological quality criteria</th></tr>
<tr><td rowspan="6">An approach for exploring and understanding meaning that individuals or groups ascribe to an issue.

Common qualitative approaches include:

Case study
In-depth exploration/explanation of issues intrinsic to an individual case. A case can be anything from a decision-making process, to a person, organisation or country and may involve multiple cases.

Ethnography
A description and interpretation of the shared cultural behaviour of a group of individuals

Framework analysis
Analysis using a structed and systematic approach to develop a thematic framework

Grounded theory
Generation of theory from data in the process of conducting research (data collection occurs first)

Narrative research
Analysis of life experiences of an individual or group

Phenomenology
Focus on the experiences and interpretation of a phenomenon encountered by individuals

Qualitative description
No specific methodology described, but involves qualitative data collection and analysis e.g. in-depth interviews and focus groups</td><td>1.1. Is the qualitative approach appropriate to answer the research question?

The qualitative approach used should be appropriate to answer the type of research question or address the issue presented (see list on the left for examples). Rationale should be provided for the use of a qualitative design and for the specific methodology.</td></tr>
<tr><td>1.2. Are the data collection methods adequate to address the research question?

This relates to data collection method, including data sources (e.g., archives, documents, interview transcripts), used to address the research question. To judge this criterion, consider whether the method of data collection (e.g., in depth interviews and/or group interviews, and/or observations) and the form of the data (e.g., tape recording, video material, diary, photo, and/or field notes) are adequate. Are they able to capture the complexity/diversity of experience and illuminate the context in sufficient detail? Clear justifications are needed if data collection methods are modified during the study.</td></tr>
<tr><td>1.3. Are the sampling methods and sample appropriate?

The study should include detailed selection criteria, a description of how sampling was undertaken and justification for the sampling strategy. Any disparity between planned and actual sample should be explained. Was data saturation discussed?</td></tr>
<tr><td>1.4. Are the findings adequately derived from the data?

This criterion is related to the data analysis used. The analytic approach should be explicit, including details of how the coding systems/conceptual frameworks were developed. There should be evidence that more than one researcher was involved in analysis and interpretation, if this is appropriate for the theoretical stance. Did the research participants have any involvement in analysis (member checking)?</td></tr>
<tr><td>1.5. Is the interpretation of the results sufficiently substantiated by data?

The interpretation of results should be supported by the data collected. For example, the quotes provided to justify the themes should be adequate and represent the essence of the described theme. The relationship between research and participants, and the researcher’s influence on analysis and interpretation should be discussed.</td></tr>
<tr><td>1.6. Is there coherence between the qualitative data sources, collection, analysis and interpretation?

There should be a clear pathway between data sources, collection, analysis and interpretation, and sufficient discussion of research processes for the reader to follow the ‘decision trail’.</td></tr>
</table>

<table>
<tr><th>2. Quantitative descriptive studies</th><th>Methodological quality criteria</th></tr>
<tr><td rowspan="5">Describe the existing distribution of variables. Not concerned with causal relationships. Used for describing the population, planning and generating hypotheses.

Common designs include:

Case report
An individual or a group with a unique/unusual outcome that is described in detail

Case series
A collection of individuals with similar characteristics used to describe an outcome

Incidence or prevalence study without comparison group
In a defined population, at one particular time, what is happening in that population e.g. frequencies of factors

Survey
Information gathered by asking people questions on a specific topic with a standardised and well-defined data collection procedure</td><td>2.1. Is the sampling strategy relevant to address the research question?

Sampling strategy refers to the way the sample was selected. There are two main categories of sampling strategies: probability sampling (involve random selection) and non-probability sampling. Depending on the research question, probability sampling might be preferable. Nonprobability sampling does not provide equal chance of being selected. To judge this criterion, consider whether the source of sample is relevant to the target population; a clear justification of the sample frame used is provided; or the sampling procedure is adequate.</td></tr>
<tr><td>2.2. Is the sample representative of the target population?

There should be a match between respondents and the target population. Indicators of representativeness include: clear description of the target population and of the sample (such as respective sizes and inclusion and exclusion criteria), reasons why certain eligible individuals chose not to participate, and any attempts to achieve a sample of participants that represents the target population.</td></tr>
<tr><td>2.3. Are the measurements appropriate?

Indicators of appropriate measurements include: the variables are clearly defined and accurately measured, the measurements are justified and appropriate for answering the research question; the measurements reflect what they are supposed to measure; validated and reliability tested measures of the outcome of interest are used, variables are measured using ‘gold standard’, or questionnaires are pre-tested prior to data collection.</td></tr>
<tr><td>2.4. Is the risk of nonresponse bias low?

To judge this criterion, consider whether the respondents and non-respondents are different on the variable of interest. This information might not always be reported in a paper. Some indicators of low nonresponse bias can be considered such as a high response rate, reasons provided for nonresponse (e.g., noncontacts vs. refusals), and statistical compensation for nonresponse, where appropriate (e.g., imputation).

The nonresponse bias is might not be pertinent for case series and case report. For these studies, complete data on the cases should be considered.</td></tr>
<tr><td>2.5. Is the statistical analysis appropriate to answer the research question?

The statistical analyses used should be clearly stated and justified in order to judge if they are appropriate for the design and research question, and whether problems with data analysis limit the interpretation of the results.</td></tr>
</table>

3. Mixed methods studies	Methodological quality criteria
A combination of qualitative and quantitative methods. Each method needs to be used rigorously in accordance to the generally acceptable criteria for that type of research. The mixed methods design should be pre-planned or established as part of an iterative research design. There needs to be integration of the qualitative and quantitative components.<br><br>Common designs include:<br><br>**Convergent design**<br>The qual and quant components are usually (but not necessarily) concomitant. The purpose is to examine the same phenomenon by interpreting these two different types of data<br><br>**Sequential explanatory design**<br>Results of phase 1 quant component informs the development of the phase 1 qual component. The quant data guides the qual sources and methods of data collection, and the qual findings contribute to the interpretation of the quant results<br><br>**Sequential exploratory design**<br>Results of phase 1 qual component informs the development of phase 2 quant component.	3.1. Is there an adequate rationale for using a mixed methods design to address the research question?<br><br>The reasons for conducting a mixed methods study should be clearly explained. Several reasons can be invoked such as to enhance or build upon qualitative findings with quantitative results and vice versa; to provide a comprehensive and complete understanding of a phenomenon or to develop and test instruments.
	3.2. Are the different components of the study effectively integrated to answer the research question?<br><br>Integration is a core component of mixed methods research. Look for information on how qualitative and quantitative phases, results, and data were integrated. For instance, how data gathered by both research methods was brought together to form a complete picture (e.g., joint displays) and when integration occurred (e.g., during the data collection-analysis or/and during the interpretation of qualitative and quantitative results).
	3.3. Are the outputs of the integration of qualitative and quantitative components adequately interpreted?<br><br>This criterion is related to meta-inference, which is defined as the overall interpretations derived from integrating qualitative and quantitative findings. Meta-inference occurs during the interpretation of the findings from the integration of the qualitative and quantitative components, and shows the added value of conducting a mixed methods study rather than having two separate studies.
	3.4. Are divergences and inconsistencies between quantitative and qualitative research adequately addressed?<br><br>When integrating the findings from the qualitative and quantitative components, divergences and inconsistencies (also called conflicts, contradictions, discordances, discrepancies, and dissonances) can be found. It is not sufficient to only report the divergences, they need to be explained. Different strategies to address the divergences have been suggested such as reconciliation, initiation, bracketing and exclusion. Rate this criterion 'Yes' if there is no divergence.
	3.5. Do the qualitative and quantitative components of the study meet the quality criteria provided in sections 1-2?<br><br>The quality of the qualitative and quantitative components should be individually appraised to ensure that no important threats to trustworthiness are present. To appraise this point, use criteria for the qualitative (1) and descriptive quantitative (2) components. The quality of both components should be high for the mixed methods study to be considered of good quality. The premise is that the overall quality of a mixed methods study cannot exceed the quality of its weakest component. For example, if the quantitative component is rated high quality and the qualitative component is rated low quality, the overall rating for this criterion will be of low quality.

Adapted from: Hong QN et al. Improving the content validity of the mixed method appraisal tool: a modified e-Delphi study. *J Clin Epidemiol* 2019; 111: 49-59

Developed for: Newington et al. Impacts of clinical academic activity outside medicine: a systematic review. https://osf.io/gj7se
