# Supplementary material for: A qualitative systematic review and thematic synthesis exploring the impacts of clinical academic activity by healthcare professionals outside medicine
Source: BMC Health Serv Res. 2021 Apr 29;21:400. doi: 10.1186/s12913-021-06354-y (PMC8082861; doi:10.1186/s12913-021-06354-y)
Supplement: Supplementary file 3 — Additional file 3. Articles excluded during full text screen and reasons for exclusion. References of the excluded articles and reason for exclusion. [file 12913_2021_6354_MOESM3_ESM.pdf]

Articles excluded during full text screening and reason for exclusion

1 Adamsen L, Larsen K, Bjerregaard L, *et al.* Danish research-active clinical nurses overcome barriers in research utilization. *Scand J Caring Sci* 2003;17:57–65. **Reason for exclusion:** Wrong activity – evidence based practice or research utilisation.

2 Andrew N, Ferguson D, McGuinness C. Promotion of a community culture in nursing research. *Nurs Times* 2008;104:30–3. **Reason for exclusion:** Wrong design – opinion piece or editorial.

3 Awaisu A, Alsalimy N. Pharmacists' involvement in and attitudes toward pharmacy practice research: A systematic review of the literature. *Res Soc Adm Pharm* 2015;11:725–48. **Reason for exclusion:** Wrong outcome – process or amount of research interest/activity.

4 Baillie L, Taylor R, Giordano R, *et al.* Evaluating the impact of scholarships. *Nurs Times* 2013;109:24–5. **Reason for exclusion:** Wrong population – not clinical academics.

5 Beal JA, Riley JM. Best organizational practices that foster scholarly nursing practice in Magnet® hospitals. *J Prof Nurs* 2019;35:187–94. **Reason for exclusion:** Wrong population – not clinical academics.

6 Bernhardt J, Shyn-Li LT. More options and better job security required in career paths of physiotherapist researchers: An observational study. *Aust J Physiother* 2008;54:135–40. **Reason for exclusion:** Wrong population – not clinical academics.

7 Birks M, Francis K, Chapman Y, *et al.* Supporting the evolution of a research culture among nurses in Malaysia. *Aust J Adv Nurs* 2009;27:89–93. **Reason for exclusion:** Wrong activity – research training.

8 Bjorn A, Hundruo Y, Wagner L. Doctoral prepared nurses in Denmark and their scientific production between 1976 and 2005. *Int Nurs Rev* 2008;55:227–33. **Reason for exclusion:** Wrong population – not clinical academics.

9 Boase S, Kim Y, Craven A, *et al.* Involving practice nurses in primary care research: The experience of multiple and competing demands. *J Adv Nurs* 2012;68:590–9. **Reason for exclusion:** Wrong population – not clinical academics.

10 Bookey-Bassett S, Bianchi A, Richards J, *et al.* Overcoming challenges to support clinician-scientist roles in Canadian Academic Health Sciences Centres. *Healthc Q* 2019;22:60–6. **Reason for exclusion:** Wrong design – opinion piece or editorial.

11 Boyd M, Gall SB, Rothpletz-Puglia P, *et al.* Characteristics and Drivers of the Registered Dietitian Nutritionist's Sustained Involvement in Clinical Research Activities: A Mixed Methods Study. *J Acad Nutr Diet* 2019;119:2099–108. **Reason for exclusion:** Wrong outcome – process or amount of research interest/activity.

12 Brim CB, Schoonover HD. Lessons learned while conducting a clinical trial to facilitate evidence-based practice: The neophyte researcher experience. *J Contin Educ Nurs* 2009;40:380–4. **Reason for exclusion:** Wrong outcome – outcome of the research, not the impact of the research activity.

13 Clancey JK. Nurse research internship program: A unique mentoring program. *J Neurosci Nurs* 2009;41:E1–6. **Reason for exclusion:** Wrong outcome – process or amount of research interest/activity.

14 Clark MT, Lewis A, Bradbury-Jones C. Critical reflections on early career research development in public health nursing. *Community Pract* 2016;89:36–9. **Reason for exclusion:** Wrong design – opinion piece or editorial.

15 Clifford C, Murray S. Pre- and post-test evaluation of a project to facilitate research development in practice in a hospital setting. *J Adv Nurs* 2001;36:685–95. **Reason for exclusion:** Wrong outcome – process or amount of research interest/activity.

16 Colbourne L, Sque M. Split personalities: role conflict between the nurse and the nurse researcher. *Nurs Times Res* 2004;9:297–304. **Reason for exclusion:** Wrong population – not clinical academics.

17 Cooke J, Nancarrow S, Hammersley V, *et al.* The 'Designated Research Team' approach to building research capacity in primary care. *Prim Heal Care Res Dev* 2006;7:78–86. **Reason for exclusion:** Wrong outcome – process or amount of research interest/activity.

18 Cruz Rivera S, Kyte DG, Aiyegbusi OL, *et al.* Assessing the impact of healthcare research: A systematic review of methodological frameworks. *PLOS Med* 2017;14:e1002370. **Reason for exclusion:** Wrong outcome – hypothesised, rather than reported impacts.

19 Dupin CM, Larsson M, Dariel O, *et al.* Conceptions of learning research: Variations amongst French and Swedish nurses. A phenomenographic study. *Nurse Educ Today* 2015;35:73–9. **Reason for exclusion:** Wrong outcome – process or amount of research interest/activity.

20 Edwards N, MacDonald JA. Building nurses' capacity in community health services. *Int J Nurs Educ Scholarsh* 2009;6:25. **Reason for exclusion:** Wrong population – not clinical academics.

21 Fullam J, Cusack E, Nugent LE. Research excellence across clinical healthcare: a novel research capacity building programme for nurses and midwives in a large Irish region. *J Res Nurs* 2018;23:692–706. **Reason for exclusion:** Wrong outcome – process or amount of research interest/activity.

22 Gillibrand WP, Burton C, Watkins GG. Clinical networks for nursing research. *Int Nurs Rev* 2002;49:188–93. **Reason for exclusion:** Wrong outcome – process or amount of research interest/activity.

23 Goode CJ, McCarty LB, Fink RM, *et al.* Mapping the organization: A bibliometric analysis of nurses' contributions to the literature. *J Nurs Adm* 2013;43:481–7. **Reason for exclusion:** Wrong population – not clinical academics.

24 Harding KE, Stephens D, Taylor NF, *et al.* Development and evaluation of an allied health research training scheme. *J Allied Health* 2010;39. **Reason for exclusion:** Wrong activity – research training.

25 Harding KE, Shields N, Whiteside M, *et al.* 'A Great First Step into Research': Stepping into research is an effective and sustainable model for research training in clinical settings a report of 6-year outcomes. *J Allied Health* 2016;45:176–82. **Reason for exclusion:** Wrong activity – evidence based practice or research utilisation.

26 Hartigan I, Cummins A, O'Connell E, *et al.* An evaluation of lecturer practitioners in Ireland. *Int J Nurs Pract* 2009;15:280–6. **Reason for exclusion:** Wrong activity – evidence based practice or research utilisation.

27 Harvey D, Plummer D, Nielsen I, *et al.* Becoming a clinician researcher in allied health. *Aust Heal Rev* 2016;40:562–9. **Reason for exclusion:** Wrong outcome – process or amount of research interest/activity.

28 Hay-Smith EJC, Brown M, Anderson L, *et al.* Once a clinician, always a clinician: a systematic review to develop a typology of clinician-researcher dual-role experiences in health research with patient-participants. *BMC Med Res Methodol* 2016;16:1–17. **Reason for exclusion:** Wrong population – includes doctors or dentists.

29 Heinemann AW. Metrics of rehabilitation research capacity. *Am J Phys Med Rehabil* 2005;84:1009–19. **Reason for exclusion:** Wrong outcome – process or amount of research interest/activity.

30 Hickey KT, Hodges EA, Thomas TL, *et al.* Initial evaluation of the Robert Wood Johnson Foundation Nurse Faculty Scholars program. *Nurs Outlook* 2014;62:394–401. **Reason for exclusion:** Wrong population – not clinical academics.

31 Hill NL, Yevchak A, Kolanowski AM, *et al.* What it takes: Perspectives from developing nurse scientists. *J Nurs Educ* 2014;53:403–9. **Reason for exclusion:** Wrong population – not clinical academics.

32 Hulcombe J, Sturgess J, Souvlis T, *et al.* An approach to building research capacity for health practitioners in a public health environment: An organisational perspective. *Aust Heal Rev* 2014;38:252–8. **Reason for exclusion:** Wrong outcome – hypothesised, rather than reported impacts.

33 Ilott I, Bury T. Research capacity: A challenge for the therapy professions. *Physiotherapy* 2002;88:194–200. **Reason for exclusion:** Wrong outcome – process or amount of research interest/activity.

34 Jones ML, Cifu DX, Backus D, *et al.* Instilling a research culture in an applied clinical setting. *Arch Phys Med Rehabil* 2013;94:S49–54. **Reason for exclusion:** Wrong design – opinion piece or editorial.

35 Kelly D, Kent B, McMahon A, *et al.* Impact case studies submitted to REF 2014: The hidden impact of nursing research. *J Res Nurs* 2016;21:256–68. **Reason for exclusion:** Wrong population – not clinical academics.

36 Landrigan PJ, Braun JM, Crain EF, *et al.* Building Capacity in Pediatric Environmental Health: The Academic Pediatric Association's Professional Development Program. *Acad Pediatr* 2019;19:421–7. **Reason for exclusion:** Wrong population – includes doctors or dentists.

37 Larkin V. Encounters in the field, challenges and negotiations in midwifery research. *Evid Based Midwifery* 2013;11:99–106. **Reason for exclusion:** Wrong population – not clinical academics.

38 Latter S, Clark JML, Geddes C, *et al.* Implementing a clinical academic career pathway in nursing; Criteria for success and challenges ahead. *J Res Nurs* 2009;14:137–48. **Reason for exclusion:** Wrong outcome – hypothesised, rather than reported impacts.

39 Liira H, Koskela T, Thulesius H, *et al.* Encouraging primary care research: Evaluation of a one-year, doctoral clinical epidemiology research course. *Scand J Prim Health Care* 2016;34:89–96. **Reason for exclusion:** Wrong population – includes doctors or dentists.

40 Marshall M, Eyre L, Lalani M, *et al.* Increasing the impact of health services research on service improvement: the researcher-in-residence model. *J R Soc Med* 2016;109:220–5. **Reason for exclusion:** Wrong population – not clinical academics.

41 Miller L, Beck C, Dowling G, *et al.* Building gerontological nursing research capacity: Research initiatives of the John A. Hartford Foundation Centers of Geriatric Nursing Excellence. *Nurs Outlook* 2006;54:189–96. **Reason for exclusion:** Wrong population – not clinical academics.

42 Munro S, Hendrix CC, Cowan LJ, *et al.* Research productivity following nursing research initiative grants. *Nurs Outlook* 2019;67:6–12. **Reason for exclusion:** Wrong population – not clinical academics.

43 NeSmith EG, Medeiros RS, Ferdinand CHB, *et al.* 'It takes a village' to raise research productivity: Impact of a Trauma Interdisciplinary Group for Research at an urban, Level 1 trauma center. *J Trauma Acute Care Surg* 2013;75:173–8. **Reason for exclusion:** Wrong population – includes doctors or dentists.

44 O'Dell K, Shah S. Evaluation of pharmacy practice residents' research abstracts and publication rate. *J Am Pharm Assoc* 2012;52:524–7. **Reason for exclusion:** Wrong population – not clinical academics.

45 van Oostveen CJ, Goedhart NS, Francke AL, *et al.* Combining clinical practice and academic work in nursing: A qualitative study about perceived importance, facilitators and barriers regarding clinical academic careers for nurses in university hospitals. *J Clin Nurs* 2017;26:4973–84. **Reason for exclusion:** Wrong outcome – hypothesised, rather than reported impacts.

46 Owen S, Ferguson K, Baguley I. The clinical activity of mental health nurse lecturers. *J Psychiatr Ment Health Nurs* 2005;12:310–6. **Reason for exclusion:** Wrong population – not clinical academics.

47 Paget SP, Caldwell PHY, Murphy J, *et al.* Moving beyond 'not enough time': factors influencing paediatric clinicians' participation in research. *Intern Med J* 2017;47:299–306. **Reason for exclusion:** Wrong population – includes doctors or dentists.

48 Redman RW, Pressler SJ, Furspan P, *et al.* Nurses in the United States with a practice doctorate: Implications for leading in the current context of healthcare. *Nurs Outlook* 2015;63:124–9. **Reason for exclusion:** Wrong population – not clinical academics.

49 Rickard CM, Williams G, Armit L, *et al.* Towards improved organisational support for nurses working in research roles in the clinical setting. A mixed method investigation. *Collegian* 2011;18:165–76. **Reason for exclusion:** Wrong outcome – process or amount of research interest/activity.

50 Ried K, Farmer EA, Weston KM. Bursaries, writing grants and fellowships: A strategy to develop research capacity in primary health care. *BMC Fam Pract* 2007;8. **Reason for exclusion:** Wrong population – includes doctors or dentists.

51 Roddam H, Cross L, Georgiou R, *et al.* Developing clinical academic researchers: Insights from practitioners and managers in nursing, midwifery and allied health. *Br J Heal Care Manag* 2019;25:282–92. **Reason for exclusion:** Wrong outcome – hypothesised, rather than reported impacts.

52 Rose RC, Prozialeck WC. American Osteopathic Association Bureau of Research. 2003;103:435–40. **Reason for exclusion:** Wrong population – not clinical academics.

53 Schirm V, Banz G, Swartz C, *et al.* Evaluation of bedside shift report: A research and evidence-based practice initiative. *Appl Nurs Res* 2018;40:20–5. **Reason for exclusion:** Wrong outcome – outcome of the research, not the impact of the research activity.

54 Shaw S, Macfarlane F, Carter Y, *et al.* Developing primary care research team: a qualitative interview study in UK general practice. *Aust J Prim Health* 2005;11:24–31. **Reason for exclusion:** Wrong population – includes doctors or dentists.

55 Spence K, Casey A. An innovative program to close the gap from research to practice: A clinical neonatal nursing fellowship. *J Neonatal Nurs* 2015;21:42–6. **Reason for exclusion:** Wrong outcome – outcome of the research, not the impact of the research activity.

56 Szymanski DM, Ozegovic JJ, Phillips JC, *et al.* Fostering scholarly productivity through academic and internship research training environments. *Train Educ Prof Psychol* 2007;1:135–46. **Reason for exclusion:** Wrong population – not clinical academics.

57 Tagney J, Haines C. Using evidence-based practice to address gaps in nursing knowledge. *Br J Nurs* 2009;18:484–9. **Reason for exclusion:** Wrong design – opinion piece or editorial.

58 Taylor J, Wright M, Hickey K, *et al.* Genome sequencing technologies and nursing: What are the roles of nurse scientists? *Nurse Res* 2017;66:198–205. **Reason for exclusion:** Wrong design – opinion piece or editorial.

59 Wenke RJ, Tynan A, Scott A, *et al.* Effects and mechanisms of an allied health research position in a Queensland regional and rural health service: A descriptive case study. *Aust Heal Rev* 2018;42:667–75. **Reason for exclusion:** Wrong population – not clinical academics.

60 Westwood G, Richardson A, Latter S, *et al.* Building clinical academic leadership capacity: sustainability through partnership. *J Res Nurs* 2018;23:346–57. **Reason for exclusion:** Wrong outcome – process or amount of research interest/activity.

61 Westwood G, Fader M, Roberts L, *et al.* How clinical academics are transforming patient care. *Heal Serv J* Published Online First: 2013. **Reason for exclusion:** Wrong design – opinion piece or editorial.

62 Whitehouse C, Smith H. The Whitehouse Report: Review of research nursing and midwifery structures, strategies and sharing of learning across the UK and Ireland in 2017. The Florence Nightingale Foundation. 2018;:1–62. **Reason for exclusion:** Wrong outcome – process or amount of research interest/activity.

63 Williams ME, Sayegh CS, Sherer S. Promoting scholarly training in a clinical psychology postdoctoral fellowship. *Train Educ Prof Psychol* 2018;12:90–5. **Reason for exclusion:** Wrong outcome – process or amount of research interest/activity.

64 Wojner Alexandrov AW, Brethour M, Cudlip F, *et al.* Postgraduate Fellowship Education and Training for Nurses: The NET SMART Experience. *Crit Care Nurs Clin North Am* 2009;21:435–49. **Reason for exclusion:** Wrong population – not clinical academics.

65 Worrall-Carter L, Snell R. Nurse academics meeting the challenges of scholarship and research. *Contemp nurse a J Aust Nurs Prof* 2003;16:40–50. **Reason for exclusion:** Wrong population – not clinical academics.

66 Vogel E. Building research capacities of dietitians: reflections by a research intern. Dietitians of Canada. 2004. **Reason for exclusion:** Wrong outcome – process or amount of research interest/activity.

67 Wenke R, Mickan S. The role and impact of research positions within health care settings in allied health: A systematic review. *BMC Health Serv Res* 2016;16. **Reason for exclusion:** Wrong design – systematic review (indivdiual papers screened).

68 Janssen J, Hale L, Mirfin-veitch B, *et al.* Building the Research Capacity of Clinical Physical Therapists Using a Participatory Action Research Approach. 2013;93:923–34. **Reason for exclusion:** Wrong outcome – process or amount of research interest/activity.

69 Perry L, Grange A, Heyman B, *et al.* Stakeholders' perceptions of a research capacity development project for nurses, midwives and allied health professionals. *J Nurs Manag* 2008;16:315–26. **Reason for exclusion:** Wrong population – not clinical academics.

70 Williams C, Miyazaki K, Borkowski D, *et al.* Research capacity and culture of the Victorian public health allied health workforce is influenced by key research support staff and location. *Aust Heal Rev* 2015;39:303–11. **Reason for exclusion:** Wrong outcome – process or amount of research interest/activity.
