# Supplementary material for: A qualitative systematic review and thematic synthesis exploring the impacts of clinical academic activity by healthcare professionals outside medicine
Source: BMC Health Serv Res. 2021 Apr 29;21:400. doi: 10.1186/s12913-021-06354-y (PMC8082861; doi:10.1186/s12913-021-06354-y)
Supplement: Supplementary file 4 — Additional file 4. Coding framework for the types of impact identified and exemplar quotes. Full listing of the theme headings with descriptors and supplementary quotes. [file 12913_2021_6354_MOESM4_ESM.pdf]

Coding framework for the types of impact identified and exemplar quotes

1. IMPACTS FOR PATIENTS	
Changes to service provision	*A survey respondent described that her project—on patients' use of cannabis for symptom relief—helped 'open up the conversation' between patients and providers on this topic and allowed providers to offer harm reduction suggestions for their patients who use cannabis* (Black et al. 2019)
	It allowed them to understand the patients' experience with the healthcare system beyond the acute care encounter, changed the way that they delivered care to vulnerable populations, and galvanized their desire to educate their peers and leadership about the specific challenges faced by this population (Brooks Carthon et al. 2017)
	The research positions were described to have contributed to clinical practice changes that improved patient and service outcomes. One manager explained, '… they've actually changed their clinical procedures and they're doing it very differently because of the research that they [with research positions] have done… (Wenke et al. 2017)
Access to evidence-based healthcare	*The impact of the study resulted in a National change in NICE [National Institute of Clinical Excellence guidelines* (Association of UK University Hospitals 2016)
	I was able to implement an evidence-based project in my unit, and immediately saw positive results in my patients, and encouraging feedback from my peers (Turkel et al. 2008)
Improved patient/carer experiences	*Patient representatives have reported high levels of satisfaction and a 'therapeutic effect' with the principal investigator role and a strong willingness to develop research alongside clinicians* (Association of UK University Hospitals 2016)
	Another participant had introduced a pre-surgery exercise programme which helped patients to feel involved in the process and was highly rated in a patient satisfaction survey (Trusson et al. 2019)
	'Nursing research is the most exciting project you can be involved with...you are looking for solutions to problems you have identified, you can actually improve patient care and satisfaction; and your satisfaction with the care you provide (Siedlecki & Albert 2017)
Research that is meaningful to patients	*If I wasn't immersed in clinical practice I would not have pursued these questions… They are questions that are important for patients and can really affect their clinical outcomes* (Nursing Midwifery and Allied Health Professsions Research Unit 2017)
	By building research capacity in the nursing workforce, the position of nurse researcher may enhance the involvement of nurses in the specialist service to collaborate with clinicians from other disciplines in designing research programs, which can truly reflect the 'complex, multidimensional nature' of cancer care and its associated problems experienced by patients and their families (Chan et al. 2010)
Improved clinical practice	*Through literature studies, the nurses felt that they acquired valuable knowledge that was applicable in everyday nurse–patient encounters* (Bäck-Pettersson et al. 2013)
	The research process teaches a number of skills and knowledge that translate into everyday clinical practice and benefit clinicians and patients at every level (Higgins et al. 2010)
	I feel more empathetic and empowered to help patients because I understand their experience in far greater detail as a researcher and clinician (Department of Health and Social Care 2012)

**2. IMPACTS FOR SERVICE PROVISION AND WORKFORCE**	
**(i) Impacts to clinical service provision**	
Improved care delivery and pathways	*They've learnt to initiate or support discussions about treatment options with other health professionals…so there are many examples where their actual practice is changing* (Wenke et al. 2017)
	Increased integration within emergency department research to improve local practice and patient care (Wenke et al. 2018)
	They also described the unique opportunity to influence from within two cultures to make meaningful changes to patient care, 'I couldn't have made that happen in five years …just as a university academic' (Wenke et al. 2017)
Translation of research into practice and evidence implementation	*Findings from the research indicated that the FLACC [Face, Legs, Activity, Cry, Consolability] scale was not reliable for this patient population so alternative scales are being reviewed for implementation throughout the organization* (Turkel et al. 2008)
	Alteration of in-service training aiming to incorporate literature searched, appraised information in order to formulate service developments (Pomeroy et al. 2003)
	Patient education in the respiratory rehabilitation classes is now more evidenced-based and I actively engender more discussion within the patient group and encourage them to lead the discussion. I now exhort other colleagues to question day-to-day practice (Department of Health and Social Care 2012)
Release of clinical staff for research	*At certain times, the pharmacists supported each other by taking additional responsibilities from others. At other times, we gave the pharmacists half-days for research rather than full days to ensure that the operational and clinical services were adequately managed. Most pharmacists still worked considerable hours after work to complete their projects. Although the pharmacy administration strongly supported the program, the support was limited at times when there was staff shortage or budget constraints* (Nazer et al. 2017)
	Recruitment to the programmes was more common from those who were established in their career. This can present its own logistical problems in terms of delivering services (Hiley et al. 2018)
	Nurse leaders were supportive of the program and honored the request for time off for several reasons. First, the research and/or evidence-based projects advanced professional nursing and resulted in practice changes in the organization… Second, replacement of the fellows or partners "time" at the bedside was allocated to the budget of Nursing Research Fellowship Program rather than the individual nursing unit (Turkel et al. 2008)
	Biggest challenge: Releasing staff from clinical duties to support submission of application, and having only a limited number of identified academics to support staff with their application (Association of UK University Hospitals 2016)
Return to clinical practice from a research role	*The secondment was an excellent way of building research/evidence skills but follow-up after a secondment or evidence-based activity needs some sort of dedicated time* (Pomeroy et al. 2003)
	When research results and outcomes point to ways in which practice and care might be changed discussion group members may confront the likelihood that their outcomes will not be taken up. This leaves nurse researchers feeling both powerless and sceptical in terms of their commitment to future research projects (Higgins et al. 2010)

**(ii). Impacts for the clinical academic workforce**	
Clinical academic career structures	*Some participants felt their managers did not necessarily comprehend that [the research fellowship] might only be the beginning of a clinical academic journey and that it could mean long term investment in that individual* (Hiley et al. 2018)
	You know staff have chosen to do bridging, PhDs, either through that route (HEE/NIHR) or through other means and the majority are really committed to clinical practice and want to maintain a presence seeing patients. That's a huge advantage to patients and patient safety but it's really hard to do that with my current budget and HR set up. So that's the bit that needs to be fixed (Hiley et al. 2018)
	Moving forward we have to look at more sustainable and integrated approaches to embedding clinical academic careers. I'm excited to hear that there's an apprenticeship framework coming out because for clinical academic careers to be truly embedded within non-medical professional career pathways, it has to be driven by the NHS. Universities get the value of clinical academics and they're on board, but for it to truly work, we need to have change within the NHS (Trusson et al. 2019)
Balancing clinical and academic components of the role	*Within the clinical setting I felt there was limited understanding of the role and confusion regarding the ownership of my clinical time* (Department of Health and Social Care 2012)
	Because of the imperative for quick results, there is a general perception that clinicians and managers do not provide sponsorship for nurses to engage in research. Managers and nurses are required constantly to meet performance indicators, prioritise waiting lists and maintain reasonable workloads. These immediate needs override the need to conduct research (Higgins et al. 2010)
	Participants reported social and cultural differences between care and science practices: whereas care is perceived as team-based, action-oriented, fast, non-flexible and informal, science is perceived as more individualistic, reflective, slow, flexible, intellectually challenging, international, and more formal in its culture and rules (e.g. those related to formal medical ethical approval procedures) (Kluijtmans et al. 2017)
Resources and support	*The only thing I keep reiterating is that we need a statistician available for [clinical academic] researchers to help run the support correct tests and double-check outputs* (Wenke et al. 2018)
	Supportive infrastructure and environment for evidence generation and utilisation is necessary to inform safe, effective and quality nursing care (Chan et al. 2010)
	The 'Expectations outlined in my job description and articulated at performance appraisal includes research but support for this is minimal (Higgins et al. 2010)
	It was a great opportunity, something that is not available in other health districts I have worked for. Most health services have ambitious goals to be involved in QI [quality improvement] or research, but few take the practical step of supporting clinicians with off-line time (Wenke et al. 2018)

3. IMPACTS TO RESEARCH PROFILE, CULTURE AND CAPACITY	
**Research profile**	
Winning research funding and other awards	*The Nursing Research Fellowship Program was successful from the perspective of the Magnet appraisers who recognized the program as a Magnet exemplar* (Turkel et al. 2008)
	A clinician commented, '… achievements would be the number of [successful] grant applications … that we probably wouldn't have even thought about previously' (Wenke et al. 2017)
Publications and presentations	*Research positions were reported to contribute to an increase in traditional research outputs, including journal publications, national and international conference presentations, and grant funding* (Wenke et al. 2017)
	Professional outcomes from the fellowship program included recognition from the ANCC [American Nurses Credentialing Center] Magnet appraisers as an exemplar, the first place poster award at the American Organization of Nurse Executives 2006 national conference, publication in a professional journal, several national poster presentations, and national and local podium presentations (Turkel et al. 2008)
**Research culture and capacity**	
Research training and support	*Although the positive impact of mentorship on the professional development of hospital pharmacists has not been described before, providing mentorship was an important element for increasing research activities among academic pharmacists* (Nazer et al. 2017)
	Improvement in research skills, knowledge and confidence: allowed through skills gained to be able to find/review relevant literature and facilitate others to do so within workplace; and provided confidence to participate in evidence-based activity (Pomeroy et al. 2003)
Research engagement and participation	*The research positions were reported to increase the number of clinicians engaging in research activity, 'Getting some of those departments that weren't doing anything to actually be doing something is a big success'. The increased activity led to a snowball effect of progressively more clinicians engaging in research, 'When I first started I think there was probably less than half a dozen people actively participating in research. I think I've got over 40 research projects currently in process now'* (Wenke et al. 2017)
	I saw another nurse conducting research and asked if I could help ...she mentored me and taught me more than I could have ever thought I could learn. . . that was eye opening. . .you can learn more by doing than by just reading...I wish I had had this opportunity earlier in my career (Siedlecki & Albert 2017)
	Following participation in the backfill period, clinicians' average total score on the RCC [Research Capacity and Culture scale] significantly increased by approximately 50%, from a mean score of 62.5 to 100.5 out of maximum 150. Fourteen out of 15 items on the RCC demonstrated a statistically significant increase from pre- to post-backfill period: Finding literature, critical review of literature, referencing system, securing funding, writing ethics application, designing questionnaires, collecting data, using data management systems, analysing qual data, analysing quant data, writing research report, integrating findings into practice, providing advice to less experienced researchers (Wenke et al. 2018)
Challenges due to a lack of research culture	*I have come across lots of negativity in pursuing a clinical academic career as a nurse who is only a few years qualified. They think to be an expert in your field you must've been qualified for like fifteen plus years. Well that's just ridiculous* (Trusson et al. 2019)
	An important aspect of the innovation that arose from the focus groups was the need to increase awareness across the hospital of staff involvement in research and recognition that this was part of their work (McKee et al. 2017)

	In the absence of a research culture, the language of doing research is unfamiliar to clinical nurses. The language used when doing research is a barrier because: It requires different learning – you have to have the knowledge of the language – we do not use research language in practice (Higgins et al. 2010)
Embedding research in practice	*When you're able to explain to the nursing staff: "This is what we're going to do [with the data]," and we show them the result, they'll come to understand the benefits, unlike filling in some standard forms that end up in the wastebin or some filing cabinet; then, people are not motivated to cooperate, and every additional request is just too much* (Kluijtmans et al. 2017)
	The survey identified new activities initiated following completion of the programmes to enhance and deliver evidence-based practice and research literacy to improve quality of care. Examples included setting up a special interest group, developing clinical guidance, and becoming a member of Q Community, a connected community supported by The Health Foundation to improve healthcare quality across the UK (Hiley et al. 2018)
	Facilitating/enhancing research skills in other clinicians: Set up journal club; Alteration of in-service training aiming to incorporate literature searched; Appraised information in order to formulate service developments (Pomeroy et al. 2003)
**Both research profile and research culture/capacity**	
Building local and external collaborations	*In addition, the research positions were described to have helped foster new internal multidisciplinary collaborations, 'It's actually through them having developed networks …that we've been able to engage in research that we may not have had opportunities to do. So medical [and] nursing colleagues have thought of us, which is great'* (Wenke et al. 2017)
	The NIHR CAT [National Institute for Health Clinical Academic Training] fellowship has been a unique opportunity to develop my skills at many different levels and include clinical practice, leadership, networking, research methodology and capacity within my NHS trust as well as developing a long-term research trajectory, collaborations and international relationships (Department of Health and Social Care 2012)
	It's actually through them having developed networks …that we've been able to engage in research that we may not have had opportunities to do. So medical [and] nursing colleagues have thought of us, which is great (Wenke et al. 2017)
Visible clinical academic pathway	*Throughout the programme, the Chief Nurse Fellows were encouraged to enhance their learning in the areas of dissemination, networking and influencing. They did this by sharing their experiences in collaboration with The Nursing Times, via blogs* (Bramley et al. 2018)
	Developing two clinical nurses into research scholars not only had a profound effect on their ability to understand, evaluate, formulate, and articulate nursing research but also has ignited a passion and commitment for nursing science that is expanding to other clinical nurses and departments within the hospital setting (Brooks Carthon et al. 2017)
	I wanted to do research, but there wasn't a strong ethos or culture in the NHS [National Health Service] for NMAHPs [nurses, midwives and allied health professionals] to do that 15 years ago,' she says. 'But now it has really started to change (Department of Health and Social Care 2012)
Attractive place work	*Highlighting successes of the fellowship internally and externally has enhanced nursing research momentum for RNs at all levels of practice. Research barriers have been eliminated and direct-care RNs are empowered to change and improve nursing practice* (Turkel et al. 2008)

**4. ECONOMIC IMPACTS**	
Funding to support the clinical academic activity	*Funding was sourced to employ a part time post-doctoral researcher with quantitative and health research experience to act as a research facilitator and provide both expertise and sharing of research workload with the nurse researchers* (McKee et al. 2017)
	The programmes are provided at no cost to participants and a single-payment grant is paid to employers (Hiley et al. 2018)
	Importantly, the [organisation's] Nursing Director provided the professional leadership necessary for sequestering ongoing funding for the nurse researcher position in a tight budgetary environment and ensuring the primacy of nursing research for this role in the multidisciplinary service context (Chan et al. 2010)
	A major advocacy role of the CNE [Chief Nurse Executive] was to secure the resources needed to create a culture where nursing research was an integral component of professional practice. The CNE secured donor funding to support nursing research activities (Turkel et al. 2008)
Funding not available or repurposed from clinical budgets	*[Clinical academics] are really committed to clinical practice and want to maintain a presence seeing patients. That's a huge advantage to patients and patient safety but it's really hard to do that with my current budget and HR set up. So that's the bit that needs to be fixed* (Hiley et al. 2018)
	In addition, participants are often required to negotiate for time and finances within their organisation or between different organisations: 'I have noticed that it is hard to conduct research in a private practice setting. Here, you're expected to meet patient turnover rates' (Kluijtmans et al. 2017)
	The pharmacy department's budget of [healthcare organisation] was able to support the travel of a few pharmacists to present their work at international conferences. The remaining pharmacists presented their research at local conferences (Nazer et al. 2017)
Cost-savings and efficiency	*The direct economic outcomes will not be known until the projects have been in place for at least 6 months* (Turkel et al. 2008)
	The potential benefits to the organisation and NHS include reduced treatment costs. Surgical treatment for this condition costs approximately £5500, compared with £300 for physiotherapy. This cost does not include sick leave in the post-surgical phase or the cost of managing surgical complications (Association of UK University Hospitals 2016)
	The data revealed numerous examples of impact resulting from participants' clinical academic careers, including the potential for substantial savings. For example, one participant's intervention removes the need for GPs' referral for physiotherapy, potentially saving 'multimillion pounds' across the NHS, and has subsequently been recognised in the NHS long-term plan (Trusson et al. 2019)
Financial implications for the clinical academic	*Clearly I think an obstacle is when you get to that high clinical level and you've got mortgages and things, it makes it very difficult to do it on a basic stipend* (Trusson et al. 2019)
	They appreciated the opportunity to study priority areas in clinical nursing while being paid (Bäck-Pettersson et al. 2013)
	With a mortgage, a baby, one on the way it was only an NIHR [National Institute for Health Research] fellowship… it was that or nothing. I'm the main breadwinner, I earn more than my husband … so that financial part was a big barrier for me. I knew that the best financial support were the NIHR ones, so I took that time to develop that application. It didn't just affect me, it would affect the whole family (Trusson et al. 2019)

5. IMPACTS ON STAFF RECRUITMENT AND RETENTION	
Maintaining a dual role	*To obtain and maintain dual positions, however, participants must overcome logistical or practical hurdles. One participant, who had recently decided to focus full-time on her PhD, said: Especially when you combine two jobs, that is pretty hard because you have to attend all of these meetings. They expect full commitment towards your doctoral research and clinical practice. Sometimes I found these hard to combine* (Kluijtmans et al. 2017)
	Biggest challenge: Dealing with issues of sustainability of appointments at the end of the [research funding] funding, particularly as the launch of the Scheme coincided with the recession and a period of austerity impacting on the NHS (Association of UK University Hospitals 2016)
	Only half of the participants in this study expressed the ambition to remain actively involved in clinical work in the future (Kluijtmans et al. 2017)
	I currently work for an NHS trust, but the lack of support makes me wonder if the only option is to not work clinically, or work bank/agency, which to me is not embracing the value clinical academics can bring to the clinical area (Trusson et al. 2019)
Support for clinical academics	*I feel like the luckiest person in the world to have received such great support, not just through having been given the time to dedicate to research* (Wenke et al. 2018)
	Participants reported benefits such as job satisfaction, increased awareness of research, enhanced skills and sense of achievement (Trusson et al. 2019)
	The scholars also reported increased job satisfaction and appreciation for how they could change practice in the institution (Brooks Carthon et al. 2017)
	26% (n=11) CAIP and one MDBP respondents have gained promotion, of which two thirds indicated that the programme had contributed to their success (Hiley et al. 2018)
	It was brilliant,' she says. 'The university dean was very encouraging. She insisted that the job should be seen as one job, with one set of objectives that fulfilled my commitments to the university and the NHS (Nursing Midwifery and Allied Health Professsions Research Unit 2017)
	Forty thousand nurses we have a national deficit of, so people can choose where they want to work. They'll be looking for organisations that are aspirational. So actually offering innovative career pathways that can intellectually challenge, but also have that direct patient care element, is going to be attractive to a lot of people (Trusson et al. 2019)

**6. IMPACTS TO KNOWLEDGE EXCHANGE**	
Dissemination	*Some nurses felt dissemination was a way to encourage other clinical nurses to become involved in research. However, many of the nurses interviewed admitted that they had not published their findings and said a major barrier to dissemination is the lack of writing skills of nurses* (Siedlecki & Albert 2017)
	Their research activities led to the preparation of five papers for publication in peer-reviewed nursing journals (most now submitted for publication or accepted) and submission of six abstracts for national and international conference presentations (Leung et al. 2012)
	All participants are regularly contacted for updates on outputs and to date more than 100 publications, 33 as first author, have been produced at last audit (Hiley et al. 2018)
	The number of publications and the multiple dissemination methods used to promote the nursing research profile of the hospital site were identified. Several participants commented on how the intervention offered the stimulus, motivation, opportunity and support to publish findings from previously completed and unpublished research untaken as part of a Master's degree. These outputs included six peer reviewed papers, two non-peer reviewed papers, ten international conference presentations, seven national conference presentations and three local conference presentations (McKee et al. 2017)
	The pharmacy department had a total of 29 abstracts presented as oral presentations or posters during the 5 years of the program. The abstracts were presented at national and international conferences (Nazer et al. 2017)
	Research scholars were involved in the dissemination of results to both internal and external audiences. Scholars participated in the development of a research abstract that was accepted for poster presentation at the Hospital of the University of Pennsylvania's annual research day. The dissemination of research findings was also delivered to individual nursing units, to [the healthcare organisation's] Translational Research Committee, and to a broader interdisciplinary audience, including hospital leadership at nursing grand rounds (Brooks Carthon et al. 2017)
Networks and collaboration	*The research scholars program has established a pivotal bridge between the University of Pennsylvania's SON [School of Nursing], CHOPR [Center for Health Outcomes and Policy Research], and [the healthcare organisation's] nurse clinicians and administrators* (Brooks Carthon et al. 2017)
	'These opportunities have helped me to develop my work network within [trust] and my own leadership skills'. In addition, opportunities to collaborate with wider stakeholder groups and departments (such as finance and procurement) gave insight into and understanding of the complexity of decision-making processes and local and national strategic priorities (Bramley et al. 2018)
	In-service training: passed on skills to colleagues within service; started a journal club; research day organised for department (Pomeroy et al. 2003)
Practice improvement	*One mentee's research project included development, delivery, and evaluation of a health promotion program. Based on evidence of the program's feasibility and successful outcomes, a team from another hospital agreed to partner to expand the health promotion program* (Leung et al. 2012)
	Importantly, the evidence developed from these studies has already influenced UK ambulance national clinical guidelines (Nursing Midwifery and Allied Health Professsions Research Unit 2017)
	An open-access resource to help professionals to deal with children in mental health crises, specifically those at risk from self-harm, had been 'disseminated nationally, not just within health, but also in social care and education settings' (Trusson et al. 2019)

**7. IMPACTS TO THE CLINICAL ACADEMIC**	
Develop networks and influence	*Invited speaker: NIHR meeting for aspiring clinical academics (February 2016, London). Invited speaker: The NIHR at 10 (pre-conference CAC workshop) (May 2016, London). Invited keynote: Health Education England across the East Midlands Scholarship event (June 2016, Leicester)* (Association of UK University Hospitals 2016)
	Being 'found' by an active clinical academic team has not only allowed her to progress her application more rapidly but has given her the support and guidance she needed to develop her personal and organisational network (Association of UK University Hospitals 2016)
	The programme has been invaluable in helping [clinical academic] understand his own potential to develop a strong academic and clinical network, and to identify support for him and his research in a number of organisations… His research helps him to influence organisational priorities, with a solid evidence base to back it up. He also acts as a fantastic role model for others in his organisation and his profession (Association of UK University Hospitals 2016)
Attitude to clinical practice	*I feel much more comfortable with reviewing the literature, collecting data, and formulating a research question. It has carried over into other areas of my practice. I was always inquisitive, but I am much more likely to research a clinical problem by going to the evidence and taking the time to find out what has been published* (Turkel et al. 2008)
	I am more confident now,' [clinical academic] says. 'The critical thinking I've learned is the key to having a different mindset and being able to think about problems in new ways' (Nursing Midwifery and Allied Health Professsions Research Unit 2017)
Develop research and leadership skills	*Other indirect benefits, which were informally expressed by APNs [Advanced Practice Nurses], were improved leadership skills and increased collaboration and consultation with clients and others on their health care team* (Leung et al. 2012)
	I don't think I'd really had any formal leadership training before that and it really made me stop and think about my interactions with people and before I had a meeting or anything like that, you kind of stop and consider what angle you were going to come at it from whereas I don't know that I did that before and I think that's changed a lot (Hiley et al. 2018)
	The opportunity allowed me to explore beyond my ward area. In terms of influencing my career I believe that I am a driven individual and would always seek out opportunities for development. The opportunity has given me an insight into research and leadership (Bramley et al. 2018)
Career opportunities	*I never saw clinical nurses actually conducting research until I came here.. .no one tells you that in nursing school.. . the only thing we learned [in nursing school] was how to read a study, and I am not sure I learned that very well'. 'No one told me that nurses actually conducted research, it always seemed to be reserved for those better educated than I was...then I saw other nurses conducting research and I thought, why not me?'* (Siedlecki & Albert 2017)
	Thanks to the Research Challenge, I decided to explore and pursue a Master's in Nursing, and I will be attending graduate school in the fall (Black et al. 2019)
	When asked about any changes to their employment/role/grade since embarking on the clinical academic pathway, almost half (31) of the 63 survey responses described positive changes, although 5 of these reported no change in their pay band. Six had been seconded but risked reverting to their lower pay band when the secondment ended (Trusson et al. 2019)
Self-fulfilment	*Being a clinical academic is often frustrating, but knowing that you have influenced*

	and improved the management of pain for many patients is worth all the effort (Department of Health and Social Care 2012)
	I'm really proud to be a nurse (Nursing Midwifery and Allied Health Professsions Research Unit 2017)
	Completing the literature search and being able to implement an intervention based on what I found has been very satisfying. I enjoyed the challenge (Turkel et al. 2008)
	When you see or hear about other clinical nurses conducting research in your organization through articles or other organizational communication, it makes you realize that it [nursing research] is valued.. .that alone feels like support...it is not always just about money and time (Siedlecki & Albert 2017)
Challenges and sacrifices	*Mentees were asked to comment on what prevented them from meeting their goals and what additional supports or aspects would have helped to meet their goals. Their comments primarily reflected mismatched expectations between time for their research project, work demands* (Leung et al. 2012)
	Specific aspects with regard to time included, making time to do the research, making time for meetings and lack of protected time for research: ' 'put under pressure, when no protected time at work.' This was further compounded by the lack of awareness with regard to the amount of time research takes, particularly the unseen elements such as cleaning data, ethics submission process and article submission and re-submission process (McKee et al. 2017)
	Most pharmacists still worked considerable hours after work to complete their projects (Nazer et al. 2017)
	Respondents who had gained clinical academic roles described having separate contracts of employment (ie, one each for NHS and academia) with separate employment and pension rights (Trusson et al. 2019)
What is takes to be a clinical academic	*I am at the end of my career, and if I had done research earlier in my career then I might have been interested, but not at this stage* (Black et al. 2019)

References

Association of UK University Hospitals. (2016). *Transforming healthcare through clinical academic roles in nursing, midwifery and allied health professions: A practical resource for healthcare provider organisations*. Accessed from: https://councilofdeans.org.uk/2016/11/transforming-healthcare-through-clinical-academic-roles-in-nursing-midwifery-and-allied-health-professions/

Bäck-Pettersson, S., Jensen, K. P., Kylén, S., Sernert, N., & Hermansson, E. (2013). Nurses' experiences of participation in a research and development programme. *Journal of Clinical Nursing*, *22*(7–8), 1103–1111

Black, A. T., Ali, S., Baumbusch, J., McNamee, K., & Mackay, M. (2019). Practice-based nursing research: Evaluation of clinical and professional impacts from a research training programme. *Journal of Clinical Nursing*, *28*(13–14), 2681–2687

Bramley, L., Manning, J. C., & Cooper, J. (2018). Engaging and developing front-line clinical nurses to drive care excellence: Evaluating the Chief Nurse Excellence in Care Junior Fellowship initiative. *Journal of Research in Nursing*, *23*(8), 678–689

Brooks Carthon, J., Holland, S., Gamble, K., Rothwell, H., Pancir, D., Ballinghoff, J., & Aiken, L. (2017). Increasing research capacity in a safety net setting through an academic clinical partnership. *Journal of*

Nursing Administration, *47*(6), 350–355

Chan, R., Gardner, G., Webster, J., & Geary, A. (2010). Building research capacity in the nursing workforce: The design and evaluation of the nurse researcher role. *Australian Journal of Advanced Nursing*, *27*(4), 62–69

Department of Health and Social Care. (2012). *Developing the Role of the Clinical Academic Researcher in the Nursing, Midwifery and Allied Health Professions.* Accessed from: https://www.gov.uk/government/publications/developing-the-role-of-the-clinical-academic-researcher-in-the-nursing-midwifery-and-allied-health-professions

Higgins, I., Parker, V., Keatinge, D., Giles, M., Winskill, R., Guest, E., Kepreotes, E., & Phelan, C. (2010). Doing clinical research: The challenges and benefits. *Contemporary Nurse*, *35*(2), 171–181

Hiley, J., Begg, C., Banks, L., Harper, L., Swift, A., & Topping, A. (2018). *West Midlands Clinical Academic Careers Programmes for Nurses, Midwives, Allied Health Professions, Pharmacists and Healthcare Scientists (NMAHPPS) Evaluation Report*. Accessed from: https://www.birminghamhealthpartners.co.uk/wp-content/uploads/2018/11/West-Mids-Clinical-Academic-Careers-Programmes-Evaluation-Report-Oct-2018-4.pdf

Kluijtmans, M., de Haan, E., Akkerman, S., & van Tartwijk, J. (2017). Professional identity in clinician-scientists: brokers between care and science. *Medical Education*, *51*(6), 645–655

Leung, D., Widger, K., Howell, D., Nelson, S., & Molassiotis, A. (2012). Mentoring advanced practice nurses in research: recommendations from a pilot program. *Canadian Oncology Nursing Journal*, *22*(1), 31–41

McKee, G., Codd, M., Dempsey, O., Gallagher, P., & Comiskey, C. (2017). Describing the implementation of an innovative intervention and evaluating its effectiveness in increasing research capacity of advanced clinical nurses: Using the consolidated framework for implementation research. *BMC Nursing*, *16*(21), doi .org/10.1186/s12912-017-0214-6

Nazer, L. H., Tuffaha, H., & Jaddoua, S. (2017). A Program to Increase Research Productivity among Hospital Pharmacists. *Journal of Pharmacy Practice*, *30*(3), 336–341

Nursing Midwifery and Allied Health Professsions Research Unit. (2017). *A clinical academic approach for nurses, midwives and allied health professionals - it's a no-brainer!* Accessed from: https://www.nmahp-ru.ac.uk/links/

Pomeroy, V. M., Tallis, R. C., & Stitt, E. (2003). Dismantling some barriers to evidenced-based rehabilitation with “hands-on” clinical research secondments. *Physiotherapy*, *89*(5), 266–275

Siedlecki, S. L., & Albert, N. M. (2017). Research-active clinical nurses: against all odds. *Journal of Clinical Nursing*, *26*(5–6), 766–773

Trusson, D., Rowley, E., & Bramley, L. (2019). A mixed-methods study of challenges and benefits of clinical academic careers for nurses, midwives and allied health professionals. *BMJ Open*, *9*, e030595 doi.org/10.1136/bmjopen-2019-030595

Turkel, M. C., Ferket, K., Reidinger, G., & Beatty, D. E. (2008). Building a nursing research fellowship in a community hospital. *Nursing Economics*, *26*(1), 26–34

Wenke, R., Ward, E. C., Hickman, I., Hulcombe, J., Phillips, R., & Mickan, S. (2017). Allied health research positions: A qualitative evaluation of their impact. *Health Research Policy and Systems*, *15*(6), doi.org/10.1186/s12961-016-0166-4 1–11

Wenke, R., Weir, K. A., Noble, C., Mahoney, J., & Mickan, S. (2018). Not enough time for research? Use of supported funding to promote allied health research activity. *Journal of Multidisciplinary Healthcare*, *11*, 269–277
